# Genome‐wide nuclear data confirm two species in the Alpine endemic land snail *Noricella oreinos* s.l. (Gastropoda, Hygromiidae)

**DOI:** 10.1111/jzs.12362

**Published:** 2020-01-24

**Authors:** Sonja Bamberger, Michael Duda, Andreas Tribsch, Elisabeth Haring, Helmut Sattmann, Oliver Macek, Matthias Affenzeller, Luise Kruckenhauser

**Affiliations:** ^1^ Central Research Laboratories Natural History Museum Vienna Vienna Austria; ^2^ Zoological Museum Center of Natural History University of Hamburg Hamburg Germany; ^3^ Third Zoological Department Natural History Museum Vienna Vienna Austria; ^4^ Department of Biosciences University of Salzburg Salzburg Austria; ^5^ Department of Integrative Zoology University of Vienna Vienna Austria

**Keywords:** Alpine endemic species, contact zone, gene flow, Hygromiidae, species delimitation

## Abstract

The Austrian endemic land snail species *Noricella oreinos* (formerly *Trochulus oreinos*) occurs in the Northeastern Calcareous Alps at high elevations. Two morphologically highly similar subspecies *N. o.* *oreinos* and *N. o. scheerpeltzi* have been described. First analyses of mitochondrial and nuclear marker sequences indicated a high genetic divergence between them. In the present study, we aimed to assess gene flow between the two subspecies which should help to re‐evaluate their taxonomic status. Sequence data and amplified fragment length polymorphism (AFLP) markers of 255 *Noricella* specimens covering the whole distribution range were analyzed. A clear geographic separation was found within the potential contact zone, the Haller Mauern mountain range. Samples of all western sites were part of the clade representing *N. o. scheerpeltzi* and almost all samples from the eastern sites clustered with *N. o. oreinos*. However, within two sampling sites of the eastern Haller Mauern, a few individuals possessed a *COI* sequence matching the *N. o. oreinos* clade whereas at the *ITS2* locus they were heterozygous possessing the alleles of both taxa. Contrary to the *ITS2* results indicating historical and/or ongoing hybridization, AFLP analyses of 202 individuals confirmed a clear separation of the two taxa congruent with the mitochondrial data. Although they occur on the same mountain range without any physical barrier, no indication of ongoing gene flow between the two taxa was found. Thus, we conclude that the two taxa are separate species *N. oreinos* and *N. scheerpeltzi*.

## Graphical abstract

We provide insights into the land snail genus *Noricella* endemic to the Northeastern Austrian Alps. Based on a nuclear multilocus dataset, a clear separation of the two subspecies of *N. oreinos* was found. This investigation included individuals from the whole distribution range focussing on the potential hybrid zone. Our finding corroborates previous results of mitochondrial DNA barcodes and genital anatomy, which showed the distinctness of both forms. Thus, we conclude that *Noricella oreinos* and *Noricella scheerpeltzi* are separate species.

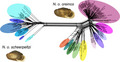

## 1 INTRODUCTION

Various concepts for defining the boundaries of species have been developed (for a general review compare Hausdorf, 2011; Mayden, 1997). A general and simple concept represents the unified species concept proposed by de Queiroz (2007). It merges the common points of alternative contemporary species concepts and defines species as “separately evolving metapopulation lineages.” Secondary properties (e.g., diagnosability, ecological divergence, monophyly, reproductive isolation) might provide further evidence for considering corresponding lineages as distinct species, but these do not constitute indispensable properties (de Queiroz, 2005, 2007). For cases of morphologically highly similar taxa, the phylogenetic relationships might appear to be challenging or even unclear. Molecular genetic analyses of mitochondrial sequences often reveal well‐differentiated clades where taxonomists fail to delimit species boundaries based on morphological and/or even ecological characters (Duda et al., 2014; Kirchner et al., 2016; Kruckenhauser et al., 2014; Proćków, Strzała, Kuźnik‐Kowalska, & Mackiewicz, 2014; Proćków, Strzała, Kuźnik‐Kowalska, Proćków, & Mackiewicz, 2017). For various reasons, researchers often abstain from more detailed investigations including markers of the nuclear genome as well as morphological characters to clarify species delimitation. Consequently, taxonomic decisions are nowadays often based on mitochondrial clades only.

**Figure 1:**
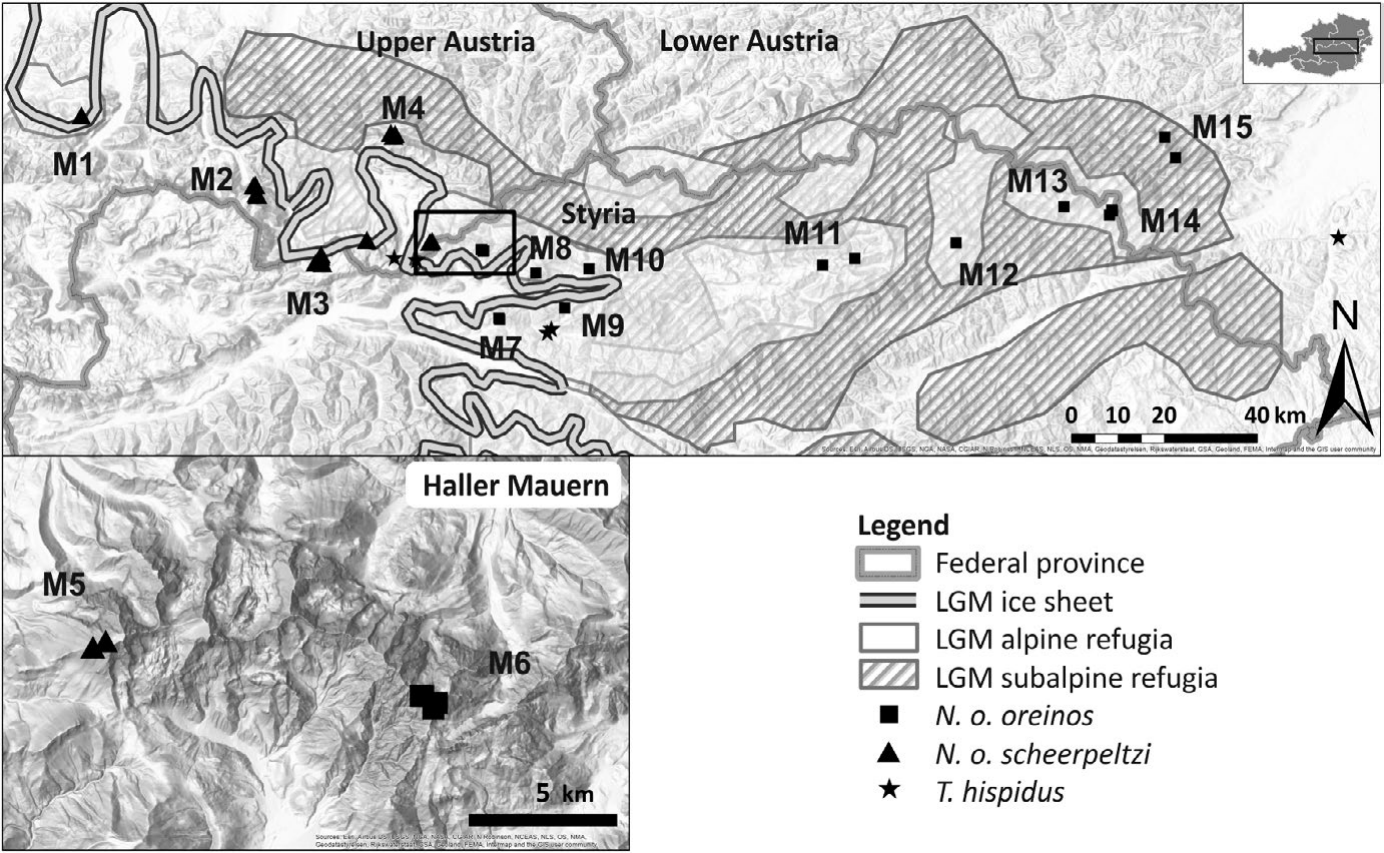
Sampling localities of *Noricella o. scheerpeltzi* (triangles), *Noricella o. oreinos* (squares) and *Trochulus hispidus* (asterisks) analyzed in this study. The Haller Mauern mountain range (rectangle) is shown in detail (bottom left). The estimated extant of the ice sheet at the Last Glacial Maximum (LGM; comp. Tribsch & Schönswetter, 2003) is indicated by a double line. This and further maps were created using ArcMap 10.4.1 (ESRI, Redlands, CA) including the Austrian administrative borders (https://www.data.gv.at/katalog/dataset/bev_verwaltungsgrenzenstichtagsdaten150000, reporting date 01.04.2017). The potential (sub)alpine LGM refugia east of the glaciated region, as postulated by Tribsch and Schönswetter (2003) for vascular plants, are displayed. Numbers correspond to the mountain regions M1–M15 defined in Table 1

The genus *Trochulus* Chemnitz, 1786 within the tribus Trochulini Lindholm, 1927 is a prominent example exhibiting high morphological variation (Proćków, 1997). Several studies attempted to address the taxonomy and to resolve the phylogenetic relationships of this genus (e.g., Dèpraz, Hausser, & Pfenninger, 2009; Duda et al., 2014; Kruckenhauser et al., 2014; Pfenninger, Hrabáková, Steinke, & Dèpraz, 2005; Proćków, 2009). Despite the existence of a high number of strongly diverged mitochondrial lineages (Kruckenhauser et al., 2014; Pfenninger et al., 2005), the systematic of *Trochulus* is still unclear in many aspects. For example, the mitochondrial lineages of the *Trochulus hispidus* complex could not be assigned to existing species names due to a lack of morphological discriminant features (Duda et al., 2014). Two taxa, initially described as subspecies of *T. hispidus* (Linnaeus, 1758) are the Austrian endemics *T. hispidus oreinos* (Wagner, 1915) and *T. hispidus scheerpeltzi* (Mikula, 1957). Later, they were proposed to form a separate species (Falkner, 1982, 1995), which was corroborated by genetic analyses that revealed high genetic distances between *T. oreinos* and *T. hispidus*. But surprisingly, also high genetic distances between the two morphologically similar subspecies *T. o. oreinos* and *T. o. scheerpeltzi* were found (see Kruckenhauser et al., 2014). The present study will concentrate on the delimitation and taxonomy of these two taxa. In a recent revision of the land snail family Hygromiidae Tyron, 1866, a comprehensive nuclear and mitochondrial dataset were analyzed including *T. oreinos* and several closely related taxa. The study revealed *T. oreinos* as well supported group within the Trochulini, which is clearly separated from the genus *Trochulus*. These results in combination with diagnostic morphological characters lead the authors to the proposal of the new monotypic genus *Noricella* for *T. oreinos* (Neiber, Razkin, & Hausdorf, 2017) which we will use further on. The species *Noricella oreinos* occurs in the Northeastern Calcareous Alps at high elevations from 1,400 m upwards and prefers rocky habitats within the subalpine and alpine ecotone (Duda, Kruckenhauser, Haring, & Sattmann, 2010; Klemm, 1974). Due to its presumed narrow ecological niche and its dispersal limitation, it is assumed to have survived the last glacial period(s) within or in close proximity to its current distribution range (Duda et al., 2014; Kruckenhauser et al., 2014). This assumption is supported by the evidence of (alpine and subalpine) Pleistocene refugia along the north‐eastern border of the Calcareous Alps, as hypothesized on basis of paleoenvironmental and geological data in combination with distribution patterns of endemic vascular plants (Tribsch & Schönswetter, 2003). *Noricella o. oreinos* and *Noricella o. scheerpeltzi* have a parapatric distribution with a possible contact area in the Haller Mauern mountain range. The easterly distributed *N. o. oreinos* is reported from Totes Gebirge, a large karst massif, to the Schneeberg mountain, which constitutes the north‐eastern margin of the distribution range, whereas the westerly distributed *N. o. scheerpeltzi* is found in Upper Austria from the Höllengebirge to Totes Gebirge and the eastern Haller Mauern (Figure 1; Klemm, 1974).

The morphological characters to distinguish the two subspecies are subtle. One conchological feature described for *N. o. scheerpeltzi*, the groove beneath the keel (Mikula, 1957), proved to be no discriminant character as it is sometimes absent (Duda et al., 2011). In addition, Duda et al. (2011) observed such a groove in several shells of the western sampling sites of *N. o. oreinos*. Yet, *N. o. oreinos* can be distinguished from *N. o. scheerpeltzi* by a genital trait: In the cross section of the penis, *N. o. oreinos* features a supplementary penial bulge and fold, which proved to be a constant character (Duda, 2012; Duda et al., 2014). Further data indicated that *N. oreinos* is monophyletic, and morphologically and genetically well distinguishable, for example, from taxa of the complex genus *Trochulus*, though some occur in close proximity (Duda et al., 2011; Kruckenhauser et al., 2014). First molecular genetic analyses were based on three mitochondrial marker sequences (partial regions of the *cytochrome c oxidase subunit 1* gene, *COI*; the *16S rRNA* and *12S rRNA* genes, *16S*, *12S*). A deep genetic split between the two subspecies was found (13.7% mean mitochondrial *p*‐distance). In addition, the nuclear marker sequence *ITS2* (*internal transcribed spacer 2*), albeit not informative to distinguish several other species of the genus *Trochulus*, separated the two subspecies clearly (0.9% mean nuclear *p*‐distance). These findings raised the question, if *N. o. scheerpeltzi* and *N. o. oreinos* represent two distinct species (Kruckenhauser et al., 2014). To infer whether there is gene flow between *N. o. oreinos* and *N. o. scheerpeltzi* and if they should be considered as separate species, we applied an additional marker system based on many nuclear loci, so‐called "Amplified Fragment Length Polymorphisms" (AFLP; Vos et al., 1995) for DNA fingerprinting.

In the current study, we used an extensive molecular genetic dataset for evaluating the phylogeographic pattern over the total distribution range and test for ongoing gene flow between the *N. oreinos* subspecies. Results from nuclear and mitochondrial DNA sequence analyses (*COI*, *ITS2*) as well as DNA fingerprinting (AFLP markers) are presented. We address the following questions: (a) Is there gene flow between the two subspecies of *N. oreinos*? If so, to what extent does the gene flow occur? (b) Do results of mitochondrial and the two nuclear DNA marker systems differ indicating asymmetric introgression? (c) Do the *N. oreinos* taxa differ in their phylogeographic structure and genetic variability? Based on the results obtained we provide a conclusion for the taxonomic status of the two taxa *N. o. oreinos* and *N. o. scheerpeltzi*.

## 2 MATERIALS AND METHODS

### 2.1 Taxon sampling

In total, 255 specimens of *N. oreinos* collected from 34 localities were analyzed (Table 1, Appendix 1). Samples have been collected in the field during previous studies (e.g., Duda et al., 2011; Kruckenhauser et al., 2014). Our sampling covers the distribution range of *N. oreinos* (comp. Klemm, 1974) with populations assigned to *N. o. oreinos* or *N. o. scheerpeltzi* by genital traits described in Duda et al. (2014). Besides *N. oreinos*, we included seven specimens of *T. hispidus* in the mitochondrial or AFLP data analyses as outgroup. Several of the localities were located in close proximity at mountains only a few hundred meters away from each other. For this reason, we defined 15 regions corresponding to various mountains (M1–M15) and assigned the localities to these regions (Figure 1, Table 1). All material is deposited in the collection of the Natural History Museum Vienna.

**Table 1:** Mountain regions and their localities

Mountain region	Locality ID	Locality	Latitude (N)	Longitude (E)	Altitude (m a.s.l.)	Number of specimens analyzed		
						*COI*	*ITS2*	AFLP
M1	12	OOE, Höllengebirge, Heumadgupf	47.81	13.71	1,677	1	1	—
M2	382	OOE, Totes Gebirge, Großer Priel, Hinterer Ackergraben	47.72	14.04	1,564	2	—	2
	383	OOE, Totes Gebirge, Großer Priel, Welser‐Hütte	47.72	14.05	1,747	3	—	2
	389	OOE, Totes Gebirge, Großer Priel, Schlund	47.71	14.05	2,284	3	—	2
	387	OOE, Totes Gebirge, Großer Priel, Fleischbanksattel	47.71	14.05	2,157	3	—	2
M3	765	ST, Totes Gebirge, Warscheneckgruppe, Hochmölbing	47.62	14.16	2,141	9	5	5
	766	ST, Totes Gebirge, Warscheneckgruppe, Hochmölbing	47.63	14.17	2,231	10	3	2
	767	ST, Totes Gebirge, Warscheneckgruppe, Hochmölbing	47.63	14.17	2,288	10	—	2
	768	ST, Totes Gebirge, Warscheneckgruppe, Hochmölbing	47.63	14.17	2,335	10	—	2
	769	ST, Totes Gebirge, Warscheneckgruppe, Angerkogel	47.62	14.18	2,072	3	—	2
	132	OOE, Totes Gebirge, Warscheneckgruppe, Toter Mann	47.65	14.26	2,028	1	—	1
M4	369	OOE, Sengsengebirge, Hoher Nock, Feichtausee	47.79	14.31	1,399	3	—	4
	351	OOE, Sengsengebirge, Hoher Nock, Hauptkar	47.78	14.31	1,704	3	—	1
	367	OOE, Sengsengebirge, Hoher Nock, Haltersitz	47.79	14.32	1,583	3	1	4
M5	443	ST, Haller Mauern west, Großer Pyhrgas, Westgrat	47.65	14.38	1,900	7	6	12
	444	ST, Haller Mauern west, Großer Pyhrgas, Westgrat summit	47.65	14.39	2,000	5	5	7
M6	783	ST, Haller Mauern east, Natterriegel	47.64	14.48	1,982	9	9 (22)	18
	782	ST, Haller Mauern east, Natterriegel	47.64	14.48	1,952	12	8 (19)	23
	784	ST, Haller Mauern east, Mittagskogel	47.63	14.49	1,948	9	8 (12)	11
	785	ST, Haller Mauern east, Mittagskogel summit	47.63	14.49	2,040	15	10 (19)	28
M7	55	ST, Gesäuse, Admonter Kalbling	47.55	14.52	2,026	6	1	5
	665	ST, Gesäuse, Admonter Kalbling, Speikboden	47.54	14.52	2,100	4	—	6
M8	781	ST, Gesäuse, Großer Buchstein	47.61	14.59	1,870	8	10 (15)	15
M9	779	ST, Gesäuse, Ennseck, Heßhütte	47.56	14.64	1,690	10	1 (2)	10
M10	399	ST, Gesäuse, Tamischbachturm	47.61	14.69	1,940	4	—	7
M11	134	ST, Hochschwab, Hochschwab, Schiestlhaus	47.62	15.14	2,179	3	1	7
	165	ST, Hochschwab, Aflenzer Staritzen, Severinkogel	47.62	15.21	2,010	1	—	—
M12	588	ST, Hohe Veitsch, summit region	47.64	15.40	1,979	3	—	3
M13	338	ST, Schneealpe, Schauerkogel	47.69	15.61	1,664	4	—	8
M14	737	ST, Rax, Wetterkogel	47.68	15.70	1,822	1	—	2
	79	NOE, Rax, Bismarcksteig	47.69	15.70	1,787	6	2	4
	448	NOE, Rax, Schlangenweg	47.69	15.70	1,600	2	—	2
M15	172	NOE, Schneeberg, Fadenwände	47.78	15.81	1,562	2	—	1
	178	NOE, Schneeberg, Waxriegel	47.76	15.83	1,873	3	—	2
Outgroup (*Trochulus hispidus*)								
	167	NOE, Bucklige Welt, Pittental, Schlattenbach	47.65	16.14	397	1	—	—
	144	ST, Gesäuse, Johnsbachtal, Kölblwirt	47.53	14.61	868	—	—	1
	145	ST, Gesäuse, Johnsbachtal, Wasserfallmauer	47.53	14.62	978	—	—	2
	237	OOE, Warscheneckgruppe, Toter Mann	47.63	14.31	810	—	—	1
	319	ST, Haller Mauern west, Großer Pyhrgas (Arlingsattel)	47.62	14.36	1,338	—	—	2

Note

The number of specimens included into the molecular genetic analyses is provided. Numbers in parentheses indicate the sequences used for *ITS2* analysis.

### 2.2 DNA extraction, amplification, and sequencing

Total genomic DNA was extracted from tissue of the foot of ethanol‐preserved specimens using the DNeasy Blood & Tissue Kit (Qiagen) following the manufacturer's instructions. Negative extraction controls (without tissue) were included for every extraction and used in the subsequent PCR amplifications.

Sequences were generated from a partial region of the mitochondrial (mt) *cytochrome c oxidase subunit I* (*COI*) gene with the PCR primers *COI*folmerfwd and *COI*schneckrev (Table S1; Duda et al., 2011; Folmer, Black, Hoeh, Lutz, & Vrijenhoek, 1994; Gittenberger, Piel, & Groenenberg, 2004). In addition, the spacer region (*ITS2*) between the nuclear *5.8S* and *28S rRNA* genes was amplified using the primer combination LSU‐1 and LSU‐3 (Table S1; Wade & Mordan, 2000). In case of more than one allele of the *ITS2* sequence, direct sequencing was unsuccessful resulting in unreadable sequences (Kruckenhauser et al., 2014). In such cases, cloning was necessary. Details on the PCR master mix, amplification conditions, and cloning procedure are given in Method S1 with the corresponding primer sequences given in Table S1.

Sequencing (both strands) was performed at LGC Genomics (Berlin, Germany) using the original PCR primers (Table S1), while for the cloned *ITS2* fragments the M13 universal primers (LGC Genomics) were used.

### 2.3 AFLP data generation

AFLP fingerprinting was performed to assess genetic diversity and structure and to identify hybrid individuals (Bensch & Åkesson, 2005; Koch, Neiber, Walther, & Hausdorf, 2016). We chose the AFLP technology for following reasons: No prior knowledge of the genome is needed (Paun & Schönswetter, 2012) and in comparison to other marker types (microsatellites, single‐nucleotide polymorphisms), AFLPs are cost efficient (Bensch & Åkesson, 2005). In addition, we expected to gain a high number of AFLP markers distributed throughout the whole genome, which ensures a sufficient resolution to examine various aspects, for example, to delimit species. For each sample, a minimum threshold of 10 ng/µl was chosen as DNA concentration to ensure the repeatability of DNA fragment generation during the AFLP procedure (Bonin et al., 2004; Vos et al., 1995). If the concentration threshold was not fulfilled, the samples were post‐processed as stated in Method S2.

With the fundamental AFLP procedure introduced by Vos et al. (1995), the AFLP protocol applied in the present study originated from Bendiksby, Tribsch, Borgen, Trávníček, and Brysting (2011). Slight modifications including primer combinations and PCR conditions are given in Method S3. As suggested by Meirmans (2015), the DNA samples were distributed randomly within and between the restriction‐ligation plates to prevent bias due to PCR performance differences. DNA fragments were separated by electrophoresis on a 48‐capillary sequencer MegaBACE 1,000 (GE Healthcare BioSciences) according to the manufacturer's instructions. The resulting electropherograms were pre‐examined visually using FRAGMENT PROFILER v1.2 (Amersham Biosciences). Binning and scoring were done using DAX 9.0 (Data Acquisition & Data Analysis Software; Van Mierlo Software Consultancy, Eindhoven, the Netherlands). The peak‐search function with subsequent manual correction was applied to locate peaks. For calibration, the ET‐ROX 400‐R MegaBACE size standard was used. The scoring bins were set manually for each fluorescent color between 88 and 405 bp fragment length. DNA fragments with peaks exceeding an intensity threshold of 300 relative fluorescent units (rfu) were scored as positive fragments. Thus, a “1” in the matrix indicates that a fragment exceeding the threshold was detected at a specific position in the electropherogram. For low‐intensity peaks (<300 rfu), background noise or non‐selective amplification was assumed (comp. Bonin et al., 2004); and therefore, these were treated as absent and scored as “0.” The resulting binary matrices of each fluorescent color were transferred to a spreadsheet for further calculations.

### 2.4 Sequence analyses

A total of 117 *COI* sequences and 107 *ITS2* sequences were generated (Appendix 1, GenBank Accession numbers MN193604–MN193721 and MN299112–MN299218). The sequences were edited in GENEIOUS 8.1.3 (Kearse et al., 2012) and combined with sequences available from former studies (Duda et al., 2011; Kruckenhauser et al., 2014). The corresponding accession numbers of the 61 *COI* sequences and 7 *ITS2* sequences downloaded from GenBank are listed in Appendix 1. Alignments were calculated using the Muscle Alignment method (Edgar, 2004) with default settings as implemented in GENEIOUS. The *ITS2* alignment was trimmed and manually adjusted in BIOEDIT 7.2.3 (Hall, 1999). Both *COI* and *ITS2* alignments are provided in Data S1 and S2. For the mt *COI* sequences, the number of unique haplotypes h was determined with the program DNASP 5.10.1 (Librado & Rozas, 2009; Rozas & Rozas, 1995). In addition, average *p*‐distances between the two *N. oreinos* taxa were calculated in MEGA7 (Kumar, Stecher, & Tamura, 2016). A neighbor‐joining (NJ) (Saitou & Nei, 1987) tree based on *p*‐distances was calculated with MEGA7 with 1,000 bootstrap replicates. The mt NJ tree was rooted with one *COI* sequence of *T. hispidus*. Median‐joining networks (Bandelt, Forster, & Röhl, 1999) were calculated in POPART 1.7 (available at http://popart.otago.ac.nz, Leigh & Bryant, 2015) for the *COI* data to visualize haplotype genealogy. From the *ITS2* sequences, a median‐joining network (Bandelt et al., 1999) was computed in NETWORK 5.0.0.0 (available at http://www.fluxus-engineering.com).

For the mt sequences, a matrix of the variable sites was generated with GENALEX version 6.502 (Peakall & Smouse, 2006, 2012). Localities represented by single specimens were excluded resulting in three datasets (*N. oreinos*: 174 *COI* sequences, *N. o. scheerpeltzi*: 74 *COI* sequences, *N. o. oreinos*: 100 *COI* sequences). Analyses of molecular variance (AMOVA; Excoffier, Smouse, & Quattro, 1992) were conducted using GENALEX to evaluate the levels of genetic diversity within and among localities as well as within the two taxa. In addition, we computed a hierarchical AMOVA for both taxa separately by assuming groups based on the geographic mountain regions as classified in Table 1. The proportion of variation explained within localities (PhiPT), among localities within groups (PhiPR), as well as among groups (PhiRT) were assessed and significance tested based on 9,999 permutations.

### 2.5 AFLP analyses

In total, 804 electropherograms were finally obtained (for the three selective primer combinations 268 electropherograms each) after accounting for capillary failures and incomplete or weak AFLP profiles (e.g., due to low DNA quality). For the whole sample set amplified with the three primer combinations, 425 different AFLP fragment lengths (hereafter called “AFLP markers”) were unambiguously scored with a bin width between 0.5 and 1.8 base pairs. Specimens that delivered repeatedly electropherograms with less than 150 scored fragments were excluded from further analyses. Twenty‐one samples (10.1% of the sample set) were repeated from the restriction‐ligation step onwards to test repeatability. The initial genotyping error rate was calculated as the observed number of phenotypic mismatches within the repeated samples divided by the total number of phenotypic comparisons (scored fragments) and averaged over all replicated samples (Bonin et al., 2004; Pompanon, Bonin, Bellemain, & Taberlet, 2005). The initial genotyping error rate was 7.5% (*SD* = 3.0%). For the analyses, all AFLP markers with an error rate of >2% were excluded. Thus, the initial dataset comprised a total of 329 AFLP markers.

Table S2 gives an overview of all datasets and subsets generated for AFLP analyses in the present study. The complete sample set (*N. oreinos*, *T. hispidus*) comprised 208 specimens and 329 AFLP markers (dataset A) and is provided in Data S3. In total, 92 AFLP markers were found exclusively in *N. oreinos* whereas 13 unique markers were found exclusively in the six *T. hispidus* specimens. Based on the initial dataset, subsets were generated for hierarchical cluster analyses with STRUCTURE. For each subset, “blank AFLP markers” were excluded to avoid influence on further calculations as these markers would not be scored for this sample sets. For example, after excluding the six specimens of *T. hispidus* from the initial dataset, for 13 AFLP markers no fragments were present. These blank AFLP markers were excluded resulting in a total number of 316 AFLP markers for the complete *N. oreinos* dataset. Of the 316 fragments present in the 202 specimens of *N. oreinos* (dataset B), 23 fragments were unique for *N. o. scheerpeltzi* and 53 fragments were found exclusively in *N. o. oreinos* specimens. The dataset C (292 AFLP markers from 114 specimens) corresponds to the supposed contact zone (Haller Mauern: M5, M6) of the two *N. oreinos* subspecies and adjacent mountain region Großer Buchstein (M8). The AFLP dataset of *N. oreinos* was subdivided according to the subspecies classification resulting in 263 AFLP markers for 50 specimens of *N. o. scheerpeltzi* (dataset D) and 293 AFLP markers for 152 specimens of *N. o. oreinos* (dataset E1). Based on the *N. o. oreinos* AFLP dataset, three further subsets were used in hierarchical cluster analyses. They contain specimens from localities of the mountain regions within the Gesäuse to the Schneeberg (M7–M15, dataset E2 containing 274 AFLP markers from 72 specimens), the Gesäuse region solely (M7–M10, dataset E3 containing 247 AFLP markers from 43 specimens), and the localities from the Hochschwab to the Schneeberg (M11‐M15, dataset E4 containing 238 AFLP markers from 29 specimens).

#### 2.5.1 Genetic diversity assessment

Levels of genetic diversity within localities were assessed by calculating the observed number of alleles (*N*_A_) and the percentage of polymorphic fragments (PPF) for the two subspecies *N. o. scheerpeltzi* and *N. o. oreinos* in GENALEX. Nei's gene diversity (D; Nei, 1987) was calculated for each locality by averaging the proportion of pairwise differences over all AFLP markers. It was computed by the use of the R function collection AFLPdat (Ehrich, 2006) in RStudio version 0.99.903 (RStudio Team, 2016) with R version 3.3.1 (R Core Team, 2016) and is supposed to be “unbiased for small sample size” (Ehrich, 2006).

#### 2.5.2 Genetic structure assessment

For a first visualization of the genetic distances between the two subspecies, a network was computed for *N. oreinos* (dataset B) in SPLITSTREE 4.14.4 (Huson & Bryant, 2006) using the neighbor‐net algorithm (Bryant & Moulton, 2004) with default settings (uncorrected observed *p*‐distances, variance: ordinary least squares). The population structure and the most likely number of genetic groups (*K*) were inferred for datasets B, C, D, and E1–E4 by the use of the Bayesian clustering approach implemented in STRUCTURE version 2.3.4 (Pritchard, Stephens, & Donnelly, 2000; Pritchard, Wen, & Falush, 2010). The admixture model within the recessive allele model was applied as ancestry model as we expected a certain extent of connectivity and gene flow between the populations. Allele frequencies were assumed to be correlated among populations (Falush, Stephens, & Pritchard, 2003). Ten replicates were calculated for each *K* using a burn‐in period of 100,000 iterations and 500,000 Monte Carlo Markov chain iterations. The most likely and biological reasonable number of *K* was inferred from two methods implemented in the STRUCTURE HARVESTER Webserver (Earl & vonHoldt, 2012). By plotting the obtained posterior probabilities of the data (mean and *SD* of ln Pr(*X*|*K*)) against the *K* values, the *K* with the highest estimated likelihood values and/or the *K* after which the slope levels off was determined, a method proposed by Pritchard et al. (2000). Additionally, the Δ*K* values (Evanno, Regnaut, & Goudet, 2005) were plotted against the *K* values to identify the number of *K* which fits the data best. The ranges of *K* chosen for STRUCTURE computations are given in Table S2. The results of both methods are illustrated in graphs in Figures S3–S4 as recommended by Gilbert et al. (2012). For each dataset and *K*, the independent runs were merged using the CLUMPAK Webserver (Kopelman, Mayzel, Jakobsson, Rosenberg, & Mayrose, 2017).

#### 2.5.3 Testing levels of genetic differentiation

Genetic differentiation was evaluated for the AFLP datasets of *N. oreinos* and the two taxa separately by computing (non‐)hierarchical Analyses of Molecular Variance (AMOVA) in GENALEX. For AMOVA calculations, we excluded localities with single specimens and used the resulting three datasets (*N. oreinos*: dataset B* containing 199 specimens, 316 AFLP marker; *N. o. scheerpeltzi*: dataset D* containing 48 specimens, 261 AFLP marker; *N. o. oreinos*: dataset E1* containing 151 specimens, 293 AFLP marker; comp. Table S2). The proportion of variation explained within localities (PhiPT), among localities within groups (PhiPR), as well as among groups (PhiRT) was calculated. Groups were defined either on the basis of the two subspecies or by allocating mountain regions to the localities as classified in Table 1. Significance was tested based on 9,999 permutations.

## 3 RESULTS

### 3.1 Sequences

#### 3.1.1 Mitochondrial sequences

The *COI* dataset (length of alignment 655 bp) comprised sequences of 178 specimens from 34 localities of *N. oreinos*. The topology of the NJ tree based on the *COI* sequences (Figure S1) was congruent with that published by Duda et al. (2011). The two subspecies were clearly separated from each other by 13.3% average genetic *p*‐distances. Within each taxon, the average genetic *p*‐distances were 0.2% for *N. o. scheerpeltzi* and 0.8% for *N. o. oreinos* (range 0%–0.9% and 0%–1.5%, respectively). In total, 30 unique haplotypes were identified with each of the two taxa represented by 15 haplotypes.

**Figure 2:**
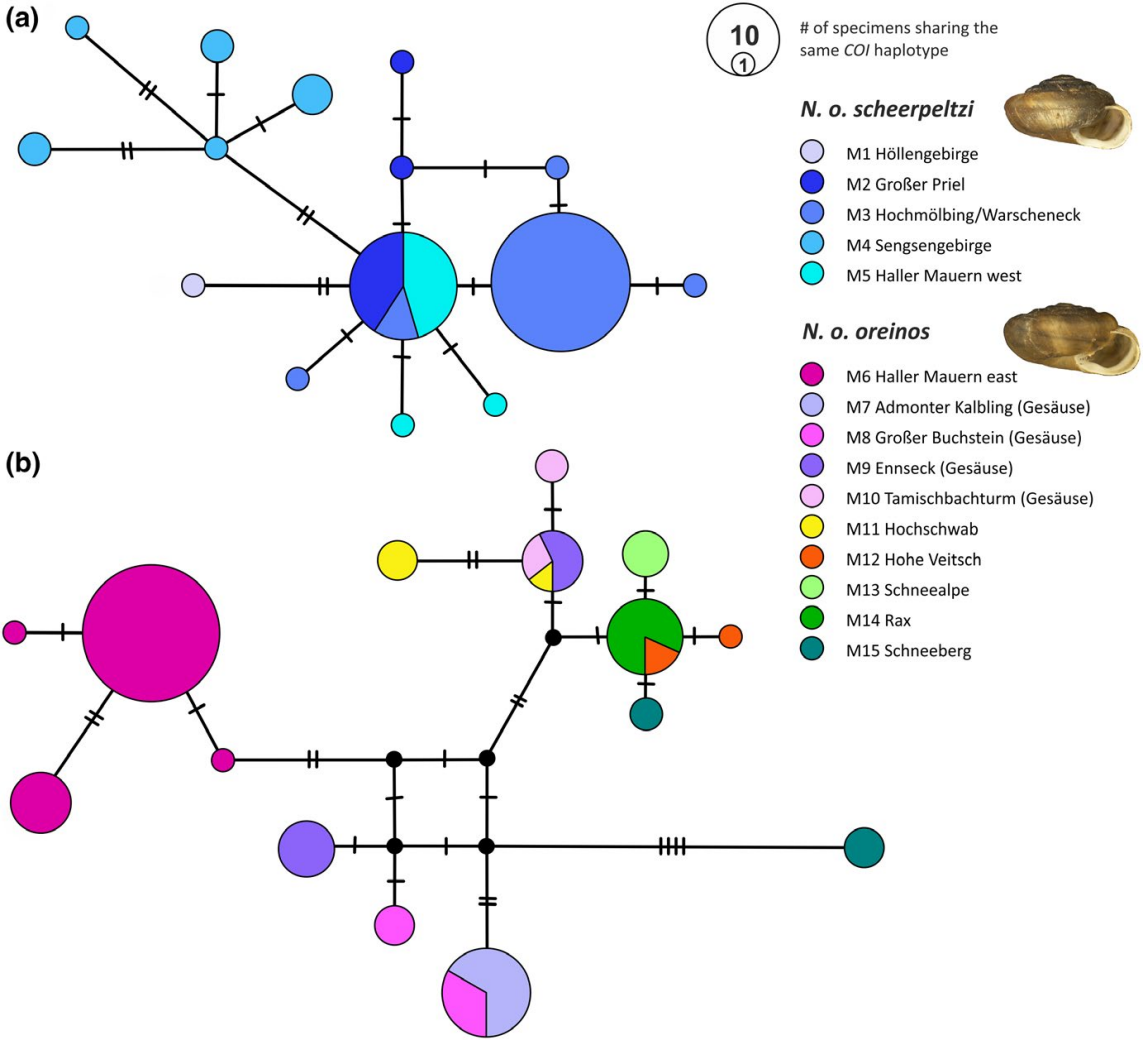
Median‐joining network of mitochondrial *COI* sequences generated from specimens of *Noricella o. scheerpeltzi* (a) and *Noricella o. oreinos* (b). The dashes on the branches indicate number of substitutions between haplotypes. The mountain regions are color‐coded (box)

In the haplotype genealogy of *N. o. scheerpeltzi* (Figure 2a), region M4 (Sengsengebirge) formed a separate haplogroup with five different haplotypes detected. The regions M2–M3–M5 (Großer Priel, Warscheneck/Hochmölbing, Großer Pyhrgas) included one shared haplotype as well as eight haplotypes separated by up to two substitutions. The haplotype detected for M1 (Höllengebirge, only one specimen analyzed) was separated by two substitutions from the M2–M3–M5 haplogroup. In the haplotype genealogy of *N. o. oreinos* (Figure 2b), *COI* sequences of the M6 region (Natterriegel and Mittagskogel, both within the eastern Haller Mauern) constituted a separate haplogroup. Haplotypes of the Gesäuse revealed high variance between and within the four mountain regions M7–M10 (Admonter Kalbling, Großer Buchstein, Ennseck, Tamischbachturm). For M7, one haplotype shared with M8 was found. For M8 and M9, the corresponding two different haplotypes were discriminated by four and six substitutions, respectively. For M10, two haplotypes were detected with one single substitution difference. From the two haplotypes detected in region M11 (Hochschwab), one was shared with a haplotype of the M9–M10 region, whereas the other haplotype differed by two substitutions. In the easternmost mountain regions M13‐M15 (Schneealpe, Rax, Schneeberg), three closely related haplotypes were found differing by a maximum of three substitutions, while one (from Schneeberg) was placed far distantly in the network (Figure 2b).

Based on the hierarchical AMOVA, 95.8% of the variance within the total *COI* sequence set was explained by distinguishing the two *N. oreinos* subspecies. When considering the two taxa separately, 72.6% and 78.4% of the genetic variance were explained by the geographic mountain regions for *N. o. scheerpeltzi* and *N. o. oreinos*, respectively. Detailed results are given in Table 2.

**Table 2:** Results of non‐hierarchical and hierarchical AMOVA derived from *COI* and amplified fragment length polymorphism (AFLP) data (*Noricella oreinos*: dataset B*, *Noricella o. scheerpeltzi*: dataset D*, *Noricella o. oreinos*: dataset E1*)

Source of variation	*COI* sequences							AFLP						
	*df*	SS	VC	TV (%)	Φ‐	Statistic	*p*‐value	*df*	SS	VC	TV (%)	Φ‐	Statistic	*p*‐value
*Noricella* sp.														
Among localities	29	3,833.089	23.016	98.5				28	4,565.100	22.675	64.3			
Within localities	144	48.957	0.340	1.5	PhiPT	0.985	.0001	170	2,136.227	12.566	35.7	PhiPT	0.643	.0001
*Noricella* sp.														
Among the two taxa	1	3,582.929	41.983	95.8	PhiRT	0.958	.0001	1	2,731.066	36.372	63.4	PhiRT	0.634	.0001
Among localities within taxa	28	250.160	1.519	3.5	PhiPR	0.817	.0001	27	1,834.034	8.416	14.7	PhiPR	0.401	.0001
Within localities	144	48.957	0.340	0.8	PhiPT	0.992	.0001	170	2,136.227	12.566	21.9	PhiPT	0.781	.0001
*N. o. scheerpeltzi*														
Among regions (M2–M5)	3	34.219	0.725	72.6	PhiRT	0.726	.0001	3	240.450	5.459	26.6	PhiRT	0.266	.0001
Among localities within regions	10	4.314	0.037	3.7	PhiPR	0.135	.0119	9	150.841	0.771	3.8	PhiPR	0.051	.0567
Within localities	60	14.224	0.237	23.7	PhiPT	0.763	.0001	35	500.626	14.304	69.7	PhiPT	0.303	.0001
*N. o. oreinos*														
Among regions (M6−15)	9	198.216	2.409	78.4	PhiRT	0.784	.0001	9	1,233.629	9.523	40.3	PhiRT	0.403	.0001
Among localities within regions	6	13.411	0.252	8.2	PhiPR	0.379	.0001	6	209.115	1.992	8.4	PhiPR	0.141	.0001
Within localities	84	34.733	0.413	13.5	PhiPT	0.865	.0001	135	1,635.601	12.116	51.3	PhiPT	0.487	.0001

Note

Significance was tested on 9,999 permutations. For each calculation, the potential source of variation, the degrees of freedom (*df*), sum of squares (SS), variance components (VC), and the percentage of total variance (TV) are indicated. The Φ‐statistic indices resulting from proportion of variation among groups (PhiRT), among localities within groups (PhiPR) and among individuals within localities (PhiPT) are specified.

#### 3.1.2 Nuclear *ITS2* sequences

**Figure 3:**
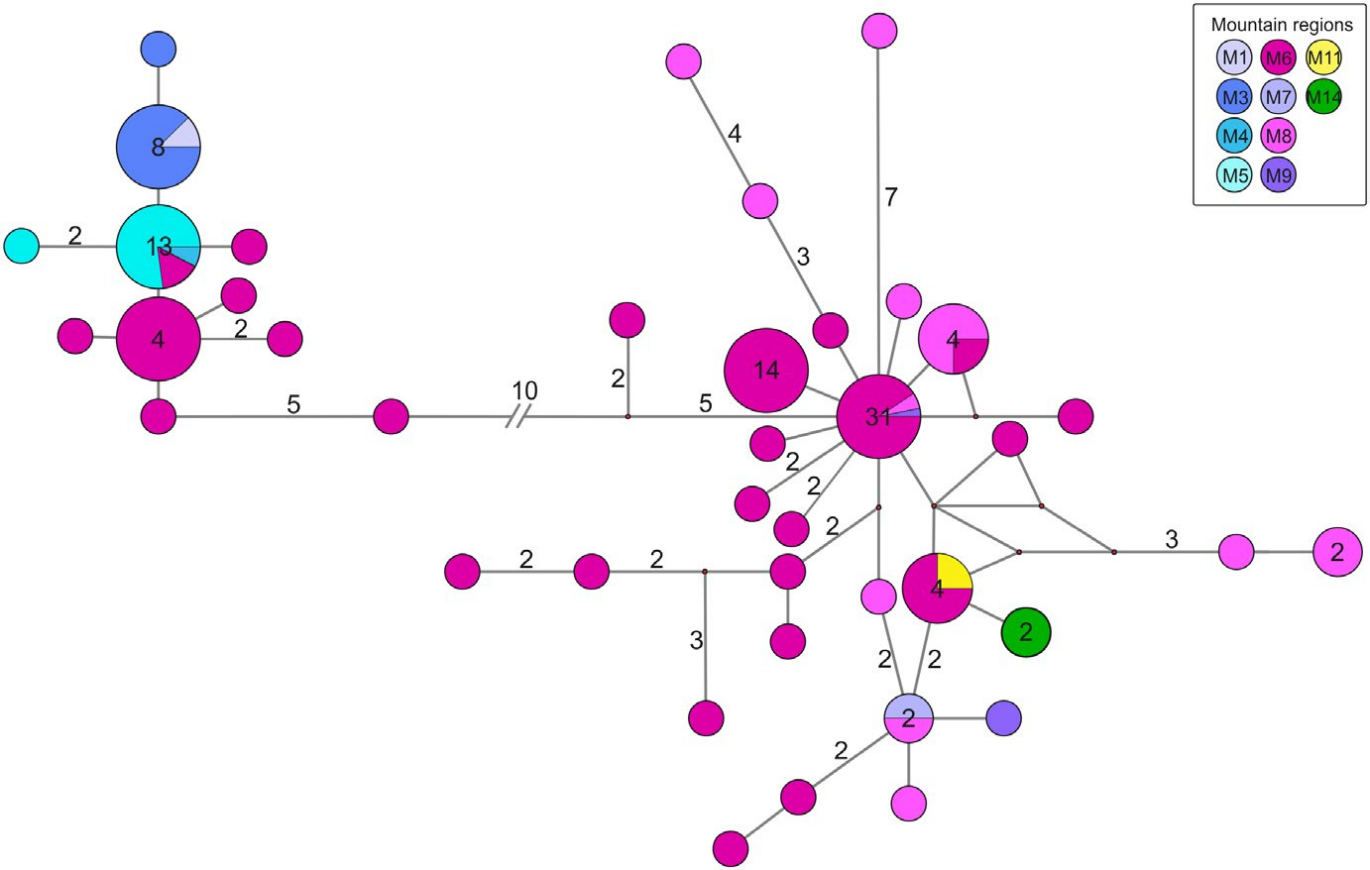
Median‐joining network of nuclear *ITS2* sequences. The dataset included 114 sequences of 51 specimens of *Noricella o. oreinos* and 20 specimens of *Noricella o. scheerpeltzi*. The size of the circles and the number within refer to the number of sequences (>1 sequences) possessing the same haplotype. The number of substitutions (>1 substitution) is given on the branches

The *ITS2* dataset (length of alignment after trimming 875 bp) included 114 sequences of 71 specimens (Appendix 1). In total, 62 specimens could be assigned to either a clade representing *N. o. oreinos* or *N. o. scheerpeltzi* on basis of the generated *ITS2* sequences. However, nine specimens from the 71 collected at the eastern Haller Mauern (M6) were heterozygous possessing the *ITS2* alleles of both *N. oreinos* taxa. The median‐joining network visualizes the *ITS2* results (Figure 3).

### 3.2 AFLP analyses

#### 3.2.1 Genetic diversity

The dataset A included 321 polymorphic AFLP markers. For the dataset D* consisting of *N. o. scheerpeltzi* specimens, 81.2% were detected and for *N. o. oreinos* (dataset E1*), there were 82.6% polymorphic fragment bands (Table S3). Considering single localities, there is a connection apparent between the number of specimens of each locality and the number of observed AFLP fragments (*N*_A_) as well as the number of polymorphic fragments (PPF). This holds especially true for localities of the Haller Mauern mountain range (*N. o. scheerpeltzi*: localities 443, 444; *N. o. oreinos*: localities 782, 784, 785) which displayed the highest values. For the latter three *N. o. oreinos* localities, the *N*_A_ values ranged between 1.06 and 1.13 and the PPF values ranged from 32.8% to 35.8% (Table S3). In contrast, the locality 134 (Hochschwab), though including only seven specimens, exhibited a PPF value of 29.7% which is similar to the maximum values. Assigning the localities to corresponding mountain regions, the highest *N*_A_ and PPF values were found likewise within the regions of the Haller Mauern, (M5 and M6: Großer Pyhrgas, Natterriegel, and Mittagskogel) for *N. o. oreinos* (Table 3). For *N. o. scheerpeltzi*, the highest value of Nei's gene diversity (D) was found within the M5 region (Großer Pyhrgas).

**Table 3:** Summary of amplified fragment length polymorphism (AFLP) variation for the geographic mountain regions calculated for both *Noricella oreinos* subspecies. For each mountain region, the number of localities *N*_Loc_, the number of specimens *N*, the mean observed number of AFLP fragments *N*_A_, the percentage of polymorphic fragments PPF, and the unbiased Nei's gene diversity (D) are given

Mountain region		*N*_Loc_	*N*	*N*_A_	PPF	*D*
*N. o. scheerpeltzi*						
M2	Großer Priel	4	8	0.977	27	0.087
M3	Warscheneck and Hochmölbling	5	13	1.218	40	0.107
M4	Sengsengebirge	2	8	0.989	27	0.102
M5	Haller Mauern west	2	19	1.379	54	0.132
*N. o. oreinos*						
M6	Haller Mauern east	4	80	1.444	57	0.093
M7	Gesäuse, Admonter Kalbling	2	11	0.993	27	0.073
M8	Gesäuse, Großer Buchstein	1	15	1.061	32	0.090
M9	Gesäuse, Ennseck	1	10	0.867	22	0.076
M10	Gesäuse, Tamischbachturm	1	7	0.891	22	0.085
M11	Hochschwab	1	7	0.983	30	0.126
M12	Hohe Veitsch	1	3	0.710	13	0.086
M13	Schneealpe	1	8	0.812	20	0.074
M14	Rax	3	8	0.799	16	0.061
M15	Schneeberg	1	2	0.628	6	0.058

#### 3.2.2 Genetic structure

**Figure 4:**
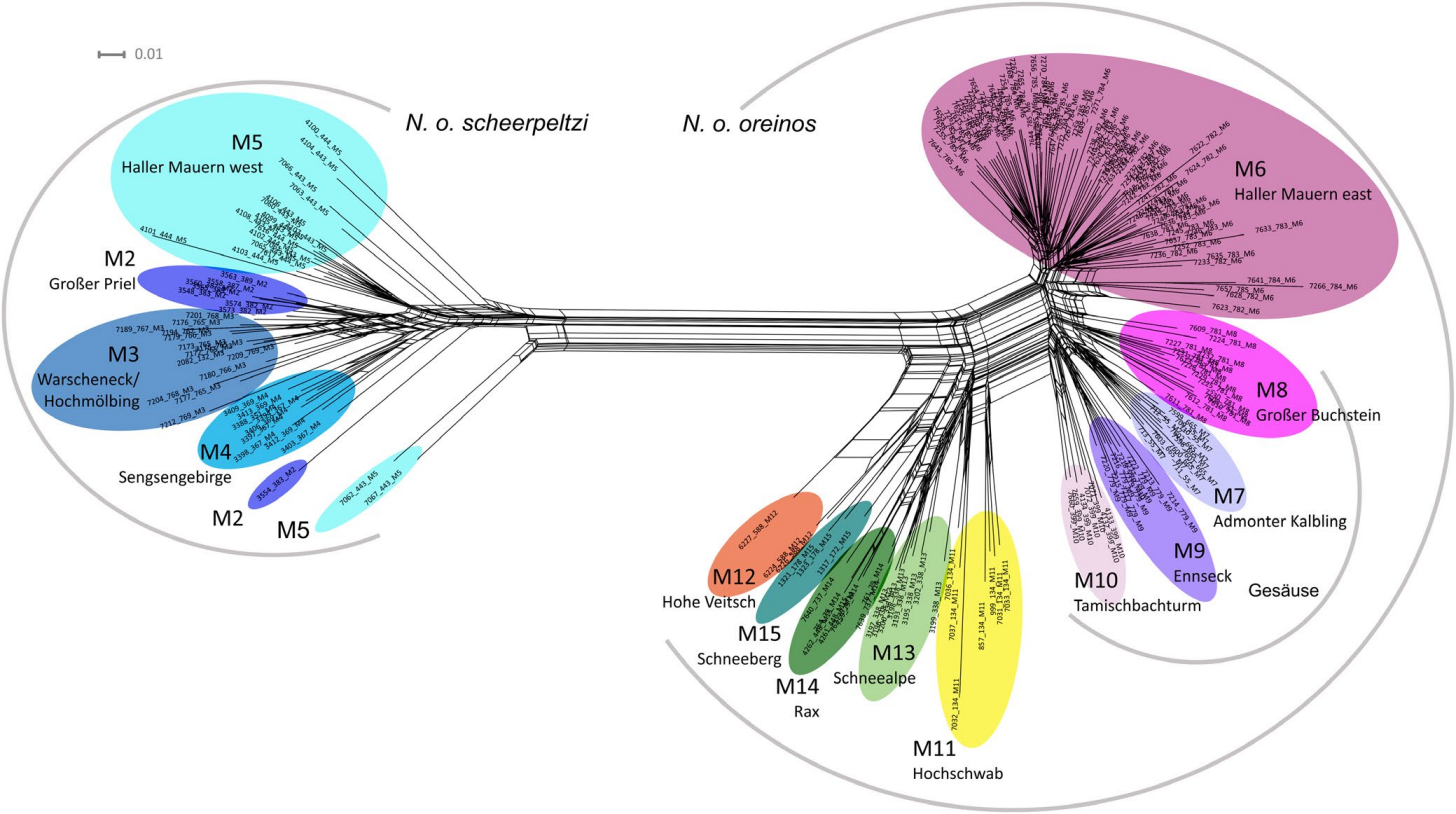
Neighbor‐net graph based on the AFLP dataset B. The different mountain regions M2–M15 are color‐coded. The scale bar indicates uncorrected *p*‐distances

**Figure 5:**
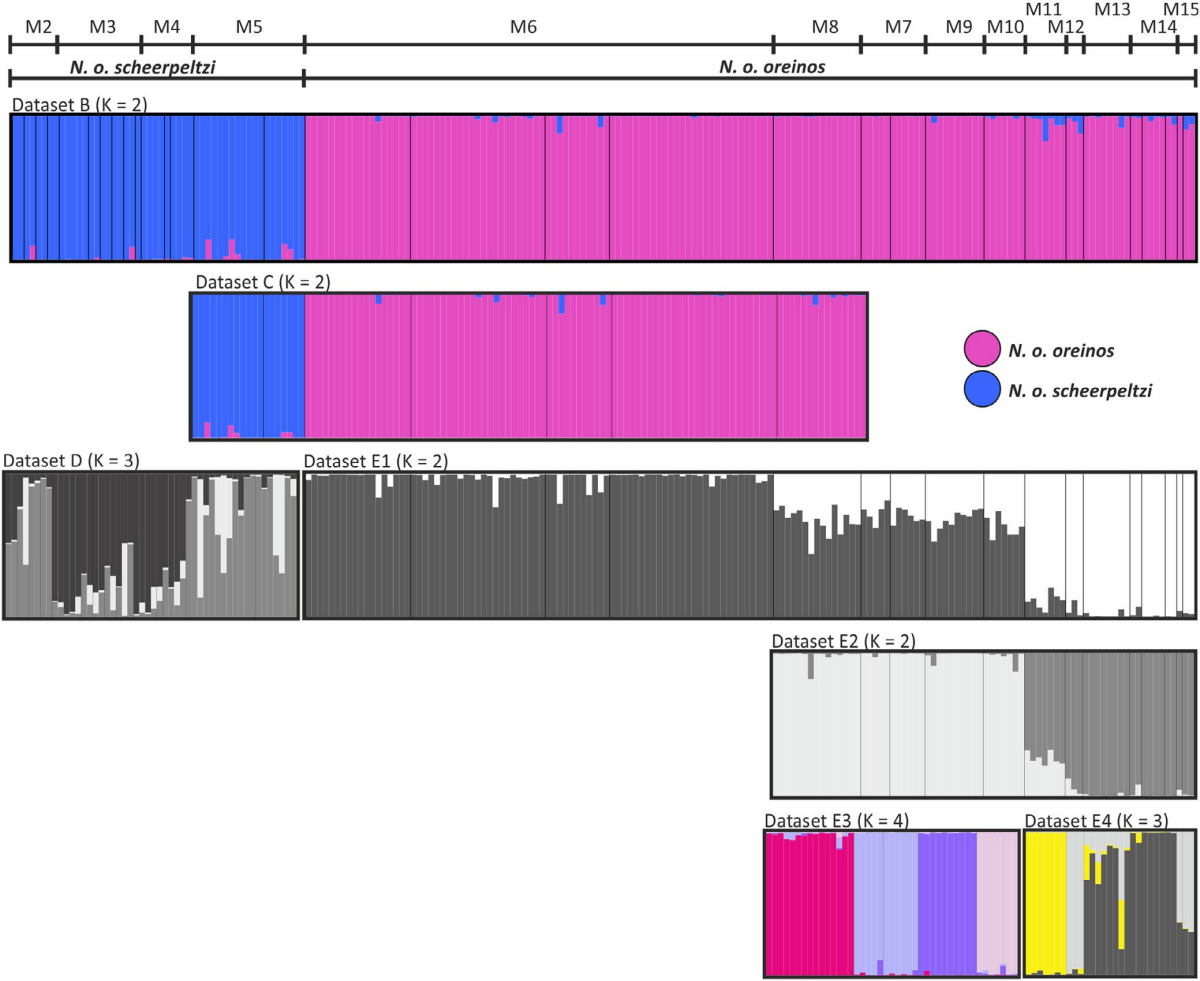
Bayesian clustering analyses based on the AFLP data. The number of clusters (*K*) which performed best due to ln Pr(*X*|*K*) estimates and Δ*K* values are given. For hierarchical STRUCTURE analysis, geographic regions were stepwise excluded resulting in data subsets for *Noricella o. oreinos*: E2 Gesäuse region to Schneeberg (M7–M15), E3 Gesäuse region (M7–M10), E4 Hochschwab to Schneeberg (M11–M15). For datasets B and C, the two clusters indicate the two *Noricella* taxa (*N. o. oreinos* pink, *N. o. scheerpeltzi* blue). Based on the datasets E3 and E4, the four Gesäuse regions as well as the Hochschwab samples correspond to separate clusters and are therefore color‐coded

The neighbor‐net network based on the AFLP dataset B (Figure 4) as well as the STRUCTURE analysis (Figure 5) confirmed the clear geographic separation of the two taxa congruent with the mt sequence data (Figure S1). Hence, in this analysis based on 316 nuclear fragments there was no indication for gene flow between *N. o. oreinos* and *N. o. scheerpeltzi* at all*.*

Likewise, *T. hispidus*, *N. o. oreinos* and *N. o.* scheerpeltzi formed three separate clusters in the scatter plots resulting from PCoA computed from the complete sample set (dataset A), with the first two axes representing 54.91% of the variation (Figure S2).

**Figure 6:**
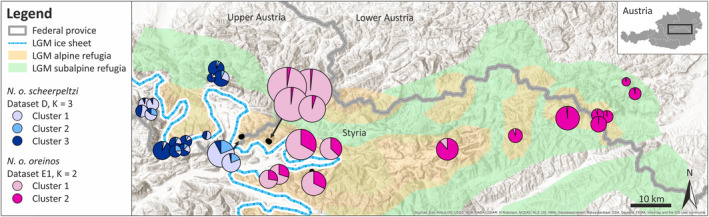
STRUCTURE results for *Noricella o. oreinos* (AFLP dataset E1, two clusters) and *Noricella o. scheerpeltzi* (AFLP dataset D, three clusters). Due to large sample sizes, the localities of the Haller Mauern east and west are dislocated in the map. For more details, see Table 1 and Figure 1

Population structure analyses were performed with the different datasets (B, C, D, E1‐E4) with the program STRUCTURE. All results are shown in Figure 5 with vertical lines representing individual AFLP profiles and with the corresponding inferred cluster ancestry of each line color‐coded. Mean estimated posterior probabilities (Pritchard et al., 2000) and Δ*K* values (Evanno et al., 2005) for each *K* computed from the different datasets are presented in Figures S3 and S4. For the complete AFLP dataset of *N. oreinos* (dataset B), the STRUCTURE analysis resulted again in two main groups representing *N. o. oreinos* and *N. o. scheerpeltzi* (Figure 5). The same result was found when examining only the specimens from the western and eastern Haller Mauern including the adjacent mountain region Großer Buchstein (M8) in the dataset C. All specimens were clearly assigned to the subspecies corresponding to the mt haplotype (Figure 5, *N. o. oreinos*: pink, *N. o. scheerpeltzi*: blue). Based on *N. o. scheerpeltzi* data solely (dataset D1), the optimal number of clusters obtained was three (comp. Figure S3). However, the graphic representation showed a more diffuse pattern: most specimens of the mountain regions M3 and M4 as well as M2 and M5 clustered together (Figure 6). For *N. o. oreinos* (dataset E1), the optimal number of clusters was two (Figure S4). While the regions M6 (Figure 5, dark gray) and M11‐M15 (Figure 5, light gray) were clearly assigned to one of the two clusters, mixed AFLP profiles were found in specimens from Gesäuse localities (M7–M10). When excluding the M6 localities (dataset E2) the analysis resulted in two clusters (Figure S4) consisting of M7–M10 (Figure 5, light gray) and M11‐15 (Figure 5, dark gray). For the dataset E3, four different clusters were detected (Figure 5) reflecting the four Gesäuse regions (M7–M10). Finally, the STRUCTURE analysis of dataset E4 revealed three clusters of which the Hochschwab (M11) was the only clearly distinguishable mountain region (Figures 5 and S4). The geographic distribution of the various clusters resulting from the STRUCTURE analyses of the datasets of *N. o. oreinos* and *N. o. scheerpeltzi* are displayed in Figure 6.

#### 3.2.3 Genetic differentiation

The AMOVA results based on the AFLP data including both *N. oreinos* taxa (dataset B*, 316 markers) revealed high levels of subdivision within localities (PhiPT = 0.643, Table 2). For this dataset, 63.4% of the total genetic variation was assigned to variation between the two taxa. The hierarchical AMOVA analysis for *N. o. scheerpeltzi* revealed a large proportion of variation due to differences among individuals within the localities (69.7%) compared to 26.6% of the variation explained among mountain regions. For *N. o. oreinos*, both the proportion of variation within localities and among mountain regions were high (51.3% versus 40.3%, respectively). A minor portion of variation among localities within regions was found for *N. o. scheerpeltzi* and *N. o. oreinos* (3.8% and 8.4%, respectively). The former was found exceeding the 5% significance level (similar to the AMOVA results for the *COI* dataset of *N. o. scheerpeltzi*, which were exceeding the 1% significance level). All other calculations were significant (*p* < .0001). Detailled AMOVA results are given in Table 2.

## 4 DISCUSSION

The starting point for the present study was the previous finding of a high genetic divergence between *N. o. oreinos* and *N. o. scheerpeltzi* based on *COI* and *ITS2* sequences (Duda et al., 2011; Kruckenhauser et al., 2014). Here, we included samples from the whole distribution range with special focus on the presumed contact zone, the Haller Mauern mountain range, to clarify the taxonomic status of the two subspecies. In addition to *COI* and *ITS2* sequences, AFLPs were investigated to assess whether there is ongoing gene flow between the two taxa. The major outcome of the current study is that there is no indication for recent gene flow between the two taxa. Hence, we regard them as separate species to which we refer further on as *Noricella oreinos* and *Noricella scheerpeltzi*.

### 4.1 Species delimitation based on genetic markers

Concerning the mitochondrial *COI* sequences, we investigated from each locality at least half the number of specimens used for AFLP analyses. The resulting phylogenetic tree included 178 *COI* sequences and resulted in two monophyletic groups, which correspond to the two *Noricella* species. At the potential contact zone, the Haller Mauern mountain range, all specimens of the western sites (Großer Pyhrgas) were assigned to the clade representing *N. scheerpeltzi*. All specimens collected at the eastern sites (Natterriegel, Mittagskogel) clustered with samples representing *N. oreinos*.

The *ITS2* is regarded as a highly variable region within the ribosomal RNA gene cluster of pulmonate land snails (Wade & Mordan, 2000). It has been proposed as a suitable nuclear marker complementary to *COI* for the evaluation of phylogenetic relationships in animals (Yao et al., 2010). Concerning gastropods, the *ITS2* marker sequence has been suggested as useful at and below the family level (Coleman & Vacquier, 2002; Wade & Mordan, 2000). The elements of the rRNA gene cluster are repeated tandemly with up to several hundred copies within one cluster. When quantifying copy numbers, high interspecific variation and additionally intraspecific divergent numbers were determined, as reviewed by Long and Dawid (1980). For several molluscs, values between 120 and 800 of repeated rRNA genes per haploid genome were reported (Long & Dawid, 1980; and references therein). In contrast to other types of (multi)gene families which undergo “divergent evolution” (e.g., hemoglobin and myoglobin) or evolve by a so‐called “birth‐and‐death” process (e.g., MHC genes), the units of the rRNA genes are meant to evolve in concert. According to this model of concerted evolution, unequal crossing over results in homogenization of the DNA sequences when mutations are absent. Therefore, similar or equal copies are expected within the gene clusters of one species compared to other species. In addition, the number of copies can vary (Nei & Rooney, 2005; and references therein). In the case of our study, the nuclear *ITS2* marker sequences delimited the two *Noricella* taxa with the exception of samples collected from the eastern Haller Mauern. At two of the four localities, there were specimens with inconsistent results: While all of the 17 specimens analyzed possessed, as expected, the mitochondrial *COI* haplotypes of *N. oreinos*, nine of them had *ITS2* alleles of both clades*.* This finding is the first indication for gene flow between the two taxa in this region. However, it has to be taken into consideration that from all other regions only 35 specimens had been analyzed with *ITS2*. The existence of specimens harboring *ITS2* alleles of both subspecies could be explained by gene flow at different time levels and/or other processes: (a) ancient (post‐glacial) gene flow within the Haller Mauern mountain range, where both taxa occur, (b) gene flow through recent sporadic hybridization, (c) ancestral polymorphisms.

Based on AFLP data generated in the present study, all specimens used in our study were assigned unambiguously to one of the two clusters representing the two *Noricella* taxa. There was no indication of recent gene flow among *N. oreinos* and *N. scheerpeltzi* at all. This in accordance with the *COI* data suggests a strong genetic differentiation between the two species*.*

In addition to the high divergence as indicated by the DNA sequence data, differences in penis anatomy (cross section of the penis) between specimens of *N. oreinos* and *N. scheerpeltzi* were reported earlier (Duda et al., 2014). Altogether, these evidences imply that recent hybridization and gene flow is rare, if it really exists at all. The non‐monophyly of the two taxa in the *ITS2* analysis can also be explained by incomplete lineage sorting of ancestral polymorphisms. Based on our data and due to the long time span since the two mitochondrial lineages have diverged, it cannot be decided if the *ITS2* polymorphisms occur due to ancient gene flow and/or ancestral polymorphisms. If gene flow did occur, we assume that these were rare events that must have taken place far enough back in time to be not detected by the AFLPs anymore. Considering the organization of the rRNA genes (including the *ITS2 region*) as a gene cluster arranged in a tandem array (Eickbush & Eickbush, 2007), it takes some time for introduced gene copies to be lost through homogenization. Thus, the different signals yielded by the various analyses (*ITS2* versus *COI* and AFLP) can be reasonably explained.

### 4.2 Geographic distribution of *Noricella*

As land snail genus endemic to the Northeastern Calcareous Alps, the *Noricella* taxa feature a confined distribution range (Duda et al., 2011; Kruckenhauser et al., 2014). With the exception of localities from the Hochschwab mountain range eastwards, *N. oreinos* and *N. scheerpeltzi* occur at the border of the presumed maximal extent of the glacial ice sheet during the Würm glacial (Figure 1). Additionally, due to the ecological and altitudinal adaptations, the two species are supposed to have survived at least the Last Glacial Maximum close to or even within its current geographic range (Duda et al., 2014; Kruckenhauser et al., 2014).

During our range‐wide sampling, specimens of *N. scheerpeltzi* were recorded from sites of the Höllengebirge, Sengsengebirge, Totes Gebirge (Großer Priel and Warscheneck/Hochmölbing), and the Großer Pyhrgas, a mountain located at the western part of the Haller Mauern mountain range within the Ennstal Alps. Therefore, *N. scheerpeltzi* showed a very narrow distribution range (five mountain ranges, M1–M5). Within the Ennstal Alps, specimens of *N. oreinos* were identified from the eastern Haller Mauern (Natterriegel, Mittagskogel) and the mountains of the Gesäuse. So far, no sympatric occurrences were recorded, but they cannot be completely ruled out. A verification is hardly possible as the high alpine mountain areas and summits within the potential contact zone, the Haller Mauern, are very difficult to access. From the Ennstal Alps eastwards, *N. oreinos* was recorded from the Hochschwab, Hohe Veitsch, Schneealpe, Rax, and the Schneeberg, which represented the easternmost edge of its distribution range. These findings are consistent with Klemm (1974). Additionally, he reported the occurrence of *N. oreinos* (formerly *N. o. oreinos*) at the Hochmölbling and the Schwarzenbergalm. Both localities are in proximity or near to mountains within the Totes Gebirge, which were assigned to *N. scheerpeltzi* based on our data. Unfortunately, in the collections of the Natural History Museum of Vienna no material of Klemm was available from these sites to compare the corresponding shells. We suspect that specimens of these sites were mistaken and correspond to *N. scheerpeltzi*. This is likely as the groove beneath the keel, a trait which was formerly used to distinguish the two taxa (Mikula, 1957), has later been proven to have minor diagnostic value (Duda et al., 2011).

In principle, our sampling covers the whole distribution range; however, some additional occurrences of the *Noricella* taxa listed by Klemm (1974) were not covered by the current study. For example, considering *N. scheerpeltzi*, specimens of the Kasberg and Schwalbenmauer are lacking. Additionally, Klemm (1974) reported *N. oreinos* from the Eisenerzer Alps from where no collected material was available. As we aimed for testing gene flow between the two subspecies, we set focus on the area where both might come in contact.

### 4.3 Patterns of genetic variability

For *N. oreinos*, geographic substructure was found concerning both the *COI* and AFLP data. Samples of the presumed contact zone at the Natterriegel and Mittagskogel (both within the Haller Mauern mountain range) appeared as a separate haplogroup in the *COI* network (Figure 2) distinct from all other sites. In addition, the AFLP data showed a separate group comprised of the Natterriegel and Mitterkogel specimens. Hence, this supports the hypothesis of one glacial refugium of *N. oreinos* within the Haller Mauern mountain range. Within the Gesäuse mountain regions, highly variable *COI* haplotypes were detected pointing to a high mitochondrial diversity within this region. Analyzed specimens were not evenly distributed throughout the whole distribution range, as we aimed to cover the whole area including interesting parts (e.g., the potential contact zone) by adequate sampling. The four Gesäuse mountain regions could be clearly delimited by the AFLP data indicating the mountains to be isolated for a longer time period. The geographic situation, that is, the position of those mountains within the Ennstal Alps right at the presumed margin of the glacier during the Last Glacial Maximum, supports the assumption of several separate glacial refugia in the Gesäuse region as indicated in the maps of van Husen (2000) and Tribsch and Schönswetter (2003). East of the Ennstal Alps, we investigated samples of the Hochschwab region (two sites). These specimens harbored shared or similar *COI* haplotypes that were also recorded from sites within the Gesäuse region. Nevertheless, the divergent AFLP patterns indicated a break from Hochschwab sites eastwards. The Hochschwab samples exhibited a higher PFF value similar to maximum values occurring. Hence, a higher level of fragment polymorphism potentially indicates higher genetic diversity within samples originating from this region. The four mountain regions from the Hohe Veitsch eastwards were represented by lower sample sizes due to the lack of available material. Nevertheless, the AFLP data still reflected the regional differentiation further east. The mitochondrial *COI* data grouped the corresponding four mountain regions (Hohe Veitsch, Schneealpe, Rax, Schneeberg) into one haplogroup except for one outlier sample from the Schneeberg, which was separated by 12 substitutions from any other haplotype detected.

Within *N. scheerpeltzi*, specimens of the Sengsengebirge formed a separate *COI* haplogroup. For the Höllengebirge, no interpretation was possible as it was represented by a single specimen only. The three remaining mountain regions (Großer Priel, Hochmölbling/Warscheneck, Haller Mauern west) were not differentiated on basis of the *COI* data: Several shared haplotypes were found without apparent geographic pattern. Concerning the AFLP data, *N. scheerpeltzi* was represented by relatively few specimens, which impedes inferences about the detailed biogeographic structure. Nevertheless, populations of *N. scheerpeltzi* appeared to be weakly structured, pointing to a complex history of extinction and recolonization within the current geographic range. One exception might represent the Sengsengebirge, where at least mitochondrial lineages indicated a separate glacial refugium of *N. scheerpeltzi*.

The genetic structuring within the genus *Noricella* resembles roughly that of another Austrian Alpine endemic snail: *Cylindrus obtusus* (Draparnaud, 1805) (Pulmonata, Helicidae). Compared to the westernmost clade of *C. obtusus, N. scheerpeltzi* has a similar distribution (Kruckenhauser et al., 2017)*.* Concerning *N. oreinos*, most eastern populations are separated in the network of mitochondrial *COI* sequences (Figure 2) as well as in the STRUCTURE analysis (Figures 5, 2 and 4) and their distribution can be compared to the eastern clade of *C. obtusus* (Kruckenhauser et al., 2017). These similarities suggest that the two species have had the same glacial refugia. A detailed comparison of work from plants (Schönswetter, Stehlik, Holderegger, & Tribsch, 2005; Tribsch & Schönswetter, 2003) and various snail species (Harl, Duda, Kruckenhauser, Sattmann, & Haring, 2014; Kirchner et al., 2016; Kruckenhauser et al., 2014, 2017) covering the same investigation area could give deeper insights into the patterns of periglacial refugia.

## 5 CONCLUSION

This study provides an extensive molecular genetic dataset of the genus *Noricella* covering the entire distribution range. By analyzing 316 AFLP markers, we found no evidence for ongoing gene flow between the two *Noricella* taxa formerly considered as the two subspecies *N. o. oreinos* and *N. o. scheerpeltzi* although ancestral hybridization was indicated by *ITS2* sequence analysis. A pronounced phylogeographic break between the western and eastern localities occurred within the *Noricella* taxa, with the Haller Mauern mountain range as contact zone where both taxa occur. Despite the pronounced genetic divergence, the two *Noricella* taxa appear conchologically and anatomically very similar, except that *N. o. oreinos* has an additional penial fold (Duda et al., 2014) and *N. o. scheerpeltzi* features a groove beneath the keel (Mikula, 1957), a trait which may also be absent or occur in morphological intermediate forms in both taxa (Duda et al., 2011). Our current knowledge points toward an old split of the two sister lineages. As a consequence, we raise the two subspecies to species status and refer to them as the separate species *Noricella scheerpeltzi* and *Noricella oreinos*. We provided here a good example on how species delimitation can be carried out on a reliable basis and hope that more studies take the effort to investigate the nature and amount of gene flow between taxa before taxonomic consequences are drawn. This would truly contribute to the understanding of the evolution and speciation in terrestrial gastropods.

## Supporting information


**Method S1.** Sequence generation.
**Method S2.** Increasing DNA concentration for AFLP generation.
**Method S3.** Amplified fragment length polymorphism.
**Table S1.** Primer combinations used for *COI* and *ITS2* amplification.
**Table S2.** List of the AFLP datasets. The number of mountain regions (Nreg), sampling localities (Nloc), specimens (N), and AFLP markers (N_AFLP_) is included with the applied programs or scripts (Splitstree, Past3, GenAlEx, AFLPdat.R, STRUCTURE). The number of *K* chosen for STRUCTURE analysis is given. The datasets D* and E1* were generated for AMOVA calculations by excluding localities with single samples from the datasets D and E. For hierarchical STRUCTURE cluster analyses, three subsets E2‐E4 were defined as specified in the text.
**Table S3.** Genetic diversity calculated for complete AFLP dataset A and the two *Noricella* taxa (datasets D* and E1*). For each locality, the number of specimens N, the mean observed number of AFLP fragments *N*_A_, the percentage of polymorphic fragments PPF and the Nei’s gene diversity (D) are given.
**Figure S1.** Neighbor‐joining tree inferred from mitochondrial *COI* sequences. Colors indicate *Noricella o. scheerpeltzi* (blue) and *Noricella o. oreinos* (pink). Bootstrap values >75% are given at the nodes. The scale bar represents 0.02 substitutions expected per aligned positions. One sequence of *T. hispidus* (black) was used as outgroup.
**Figure S2.** Scatter plots of the first and second axis of the PCoA based on Jaccard similarity estimates calculated for the AFLP dataset A. In total, 50 individuals of *Noricella o. scheerpeltzi* (blue dots), 152 individuals of *Noricella o. oreinos* (pink dots), and six individuals of *T. hispidus* used as outgroup (black triangles) were analyzed. The axes are scaled by the square root of the Eigenvalues. Eigenvalues and variance represented by the first two axes are indicated at the axes (a) and eigenvalues of the first 10 axes are illustrated (b). Specimens of the potential contact zone (M6 region, Haller Mauern east) are drawn with dark pink dots. The specimens found heterozygous, possessing the *ITS2* alleles of both taxa (violet colored), are clustering within the M6 region (black arrow).
**Figure S3.** Mean and SD values of the ln Pr(*X*|*K*) estimates and the Δ*K* values for each *K* as computed in STRUCTURE for the datasets B‐D.
**Figure S4.** Mean and SD values of the ln Pr(*X*|*K*) estimates and the Δ*K* values for each *K* as computed in STRUCTURE for the datasets E1‐E4.

**Data S1.** *COI* alignment.

**Data S2.** *ITS2* alignment.

**Data S3.** *AFLP dataset A*.

## Appendix 1

List of 262 specimens used for *COI*, *ITS2* and AFLP analyses. For each specimen, the individual ID (indID; corresponding to the NHMW 109000/Al/#####/### collection numbers), the mountain region (mregID) and the locality ID are given. The GenBank accession numbers of the *COI* and *ITS2* sequences generated for this study are printed in bold.

**Table:**

inID	mregID	Locality ID	*COI*	*ITS2*	AFLP
*Noricella o. oreinos*					
01062/707	M7	55	HQ204396	KJ151725	
01062/708	M7	55	HQ204397		
01062/709	M7	55	HQ204398		x
01062/710	M7	55	HQ204399		x
01062/711	M7	55	HQ204400		x
01062/712	M7	55	HQ204401		x
01141/713	M7	55			x
01063/754	M14	79	HQ204408		x
01063/757	M14	79	HQ204409	KJ151729	
01063/758	M14	79	HQ204410		
01063/759	M14	79	HQ204411	KJ151730	x
01063/760	M14	79	HQ204412		
01063/761	M14	79	HQ204413		x
01063/762	M14	79			x
01064/857	M11	134	HQ204420		x
01064/999	M11	134	HQ204422	KJ151734	x
01064/1000	M11	134	HQ204421		
01142/1316	M15	172	HQ204417		
01142/1317	M15	172	HQ204418		x
01065/1320	M15	178	HQ204405		
01065/1321	M15	178	HQ204406		x
01065/1323	M15	178	HQ204407		x
01185/1831	M11	165	HQ204419		
01066/3193	M13	338	HQ204414		x
01066/3194	M13	338	HQ204415		
01066/3195	M13	338	HQ204416		x
01066/3196	M13	338			x
01066/3197	M13	338	**MN193717**		x
01066/3198	M13	338			x
01066/3199	M13	338			x
01066/3200	M13	338			x
01066/3202	M13	338			x
01067/4132	M10	399	HQ204402		x
01067/4133	M10	399	HQ204403		x
01067/4134	M10	399	HQ204404		x
01109/4261	M14	448	KJ151487		x
01109/4262	M14	448	KJ151488		x
01068/6224	M12	588	KJ151512		x
01068/6226	M12	588	KJ151513		x
01068/6227	M12	588	KJ151514		x
01458/7031	M11	134			x
01458/7032	M11	134			x
01458/7033	M11	134			x
01458/7036	M11	134			x
01458/7037	M11	134			x
01067/7071	M10	399			x
01067/7072	M10	399			x
01512/7213	M9	779	**MN193648**		x
01512/7214	M9	779	**MN193649**		x
01512/7215	M9	779	**MN193650**		x
01512/7216	M9	779	**MN193651**		x
01512/7217	M9	779	**MN193652**		x
01512/7218	M9	779	**MN193653**		x
01512/7219	M9	779	**MN193654**		x
01512/7220	M9	779	**MN193655**		x
01512/7221	M9	779	**MN193656**		x
01512/7222	M9	779	**MN193657**	**MN299121** **‐** **MN299122**	x
01161/7223	M8	781	**MN193658**	**MN299123** **‐** **MN299124**	
01161/7224	M8	781		**MN299125**	x
01161/7225	M8	781		**MN299126**	x
01161/7226	M8	781	**MN193659**	**MN299127**	x
01161/7227	M8	781	**MN193660**	**MN299128**	x
01161/7228	M8	781	**MN193661**	**MN299129**	x
01161/7229	M8	781	**MN193662**	**MN299130**	x
01161/7230	M8	781	**MN193663**	**MN299131** **‐** **MN299133**	x
01161/7231	M8	781	**MN193664**	**MN299134**	x
01161/7232	M8	781	**MN193665**	**MN299135** **‐** **MN299137**	x
01168/7233	M6	782	**MN193666**	**MN299138** **‐** **MN299139**	x
01168/7234	M6	782	**MN193667**	**MN299140** **‐** **MN299144**	x
01168/7235	M6	782	**MN193668**	**MN299145** **‐** **MN299146**	x
01168/7236	M6	782	**MN193669**		x
01168/7237	M6	782	**MN193670**	**MN299147**	x
01168/7238	M6	782	**MN193671**	**MN299148**	x
01168/7239	M6	782	**MN193672**		x
01168/7240	M6	782	**MN193673**	**MN299149**	x
01168/7241	M6	782	**MN193674**	**MN299150** **‐** **MN299151**	x
01168/7242	M6	782	**MN193675**	**MN299152** **‐** **MN299156**	x
01166/7243	M6	783	**MN193676**	**MN299157**	x
01166/7244	M6	783	**MN193677**	**MN299158** **‐** **MN299164**	x
01166/7245	M6	783	**MN193678**	**MN299165**	x
01166/7246	M6	783	**MN193679**	**MN299166** **‐** **MN299167**	x
01166/7247	M6	783	**MN193680**	**MN299168** **‐** **MN299171**	x
01166/7248	M6	783	**MN193681**	**MN299172**	x
01166/7249	M6	783	**MN193682**	**MN299173**	x
01166/7250	M6	783	**MN193683**	**MN299174**	x
01166/7251	M6	783	**MN193684**	**MN299175** **‐** **MN299178**	x
01166/7252	M6	783			x
01164/7253	M6	785	**MN193685**	**MN299179** **‐** **MN299180**	x
01164/7254	M6	785	**MN193686**	**MN299181** **‐** **MN299183**	x
01164/7255	M6	785	**MN193687**	**MN299184** **‐** **MN299185**	x
01164/7256	M6	785	**MN193688**	**MN299186** **‐** **MN299187**	x
01164/7257	M6	785	**MN193689**	**MN299188**	x
01164/7258	M6	785	**MN193690**	**MN299189**	x
01164/7259	M6	785	**MN193691**	**MN299190** **‐** **MN299191**	x
01164/7260	M6	785	**MN193692**	**MN299192** **‐** **MN299193**	x
01164/7261	M6	785	**MN193693**	**MN299194** **‐** **MN299195**	x
01164/7262	M6	785	**MN193694**	**MN299196** **‐** **MN299197**	x
01165/7263	M6	784	**MN193695**	**MN299198**	
01165/7264	M6	784	**MN193696**	**MN299199** **‐** **MN299200**	x
01165/7265	M6	784	**MN193697**	**MN299201**	x
01165/7266	M6	784	**MN193698**	**MN299202**	x
01165/7267	M6	784	**MN193699**	**MN299203** **‐** **MN299204**	x
01165/7268	M6	784	**MN193700**	**MN299205**	x
01165/7269	M6	784	**MN193701**	**MN299206** **‐** **MN299207**	x
01165/7270	M6	784			x
01165/7271	M6	784	**MN193702**	**MN299208** **‐** **MN299209**	x
01165/7272	M6	785	**MN193703**		x
01162/7598	M7	665	**MN193704**		x
01162/7599	M7	665	**MN193705**		x
01162/7600	M7	665	**MN193706**		x
01162/7601	M7	665	**MN193707**		x
01162/7602	M7	665			x
01162/7603	M7	665			x
01161/7608	M8	781			x
01161/7609	M8	781			x
01161/7610	M8	781			x
01161/7611	M8	781			x
01161/7612	M8	781			x
01164/7614	M6	785			x
01164/7615	M6	785			x
01168/7618	M6	782			x
01168/7619	M6	782			x
01168/7620	M6	782			x
01168/7621	M6	782			x
01168/7622	M6	782			x
01168/7623	M6	782			x
01168/7624	M6	782			x
01168/7625	M6	782			x
01168/7626	M6	782			x
01168/7627	M6	782			x
01168/7628	M6	782	**MN193708**		x
01168/7629	M6	782	**MN193709**		x
01168/7630	M6	782			x
01166/7631	M6	783			x
01166/7632	M6	783			x
01166/7633	M6	783			x
01166/7634	M6	783			x
01166/7635	M6	783			x
01166/7636	M6	783			x
01166/7637	M6	783			x
01166/7638	M6	783			x
01167/7639	M14	737			x
01167/7640	M14	737	**MN193710**		x
01165/7641	M6	784			x
01165/7642	M6	784			x
01164/7643	M6	785	**MN193711**		x
01164/7644	M6	785	**MN193712**		x
01164/7645	M6	785	**MN193713**		x
01164/7646	M6	785	**MN193714**		x
01164/7647	M6	785	**MN193715**		x
01164/7648	M6	785			x
01164/7649	M6	785			x
01164/7650	M6	785			x
01164/7651	M6	785			x
01164/7652	M6	785			x
01164/7653	M6	785			x
01164/7654	M6	785			x
01164/7655	M6	785			x
01164/7656	M6	785			x
01164/7657	M6	785			x
01164/7658	M6	785			x
01067/7659	M10	399			x
01067/7660	M10	399	**MN193716**		x
*Noricella o. scheerpeltzi*					
01029/767	M1	12	HQ204394	KJ151731	
01069/2082	M3	132	HQ204395		x
01070/3384	M4	351	HQ204386		
01070/3385	M4	351	HQ204391		
01070/3386	M4	351	HQ204389		
01070/3388	M4	351			x
01071/3394	M4	367	HQ204390		x
01071/3395	M4	367	HQ204393		
01071/3396	M4	367	HQ204392	KJ151742	
01071/3397	M4	367			x
01071/3398	M4	367			x
01071/3403	M4	367			x
01072/3404	M4	369	KJ151442		
01072/3405	M4	369	HQ204387		
01072/3406	M4	369	HQ204388		x
01072/3409	M4	369			x
01072/3412	M4	369			x
01072/3413	M4	369			x
01073/3546	M2	383	HQ204370		
01073/3547	M2	383	HQ204371		
01073/3548	M2	383	HQ204372		x
01073/3554	M2	383			x
01110/3556	M2	387	HQ204373		
01110/3557	M2	387	HQ204374		
01110/3558	M2	387	HQ204375		x
01110/3560	M2	387			x
01111/3563	M2	389	HQ204376		x
01111/3564	M2	389	HQ204377		
01111/3565	M2	389	KJ151446		x
01074/3573	M2	382	HQ204378		x
01074/3574	M2	382	HQ204379		x
01075/4099	M5	444	**MN193718**	**MN299210**	x
01075/4100	M5	444	**MN193719**	**MN299211**	x
01075/4101	M5	444	HQ204380	**MN299212**	x
01075/4102	M5	444	HQ204381	**MN299213**	x
01075/4103	M5	444	HQ204382	**MN299214**	x
01076/4104	M5	443	HQ204383	**MN299215**	x
01076/4105	M5	443	HQ204384	**MN299216**	x
01076/4106	M5	443	HQ204385	KJ151745	x
01076/4107	M5	443	**MN193720**	**MN299217**	x
01076/4108	M5	443	**MN193721**	**MN299218**	x
01076/7060	M5	443	**MN193604**		x
01076/7061	M5	443	**MN193605**		x
01076/7062	M5	443			x
01076/7063	M5	443		**MN299112**	x
01076/7065	M5	443			x
01076/7066	M5	443			x
01076/7067	M5	443			x
01163/7170	M3	765	**MN193606**	**MN299113**	
01163/7171	M3	765	**MN193607**	**MN299114**	
01163/7172	M3	765	**MN193608**	**MN299115**	x
01163/7173	M3	765	**MN193609**	**MN299116**	x
01163/7174	M3	765	**MN193610**		
01163/7175	M3	765	**MN193611**	**MN299117**	x
01163/7176	M3	765	**MN193612**		x
01163/7177	M3	765	**MN193613**		x
01163/7178	M3	765	**MN193614**		
01508/7179	M3	766	**MN193615**		x
01508/7180	M3	766	**MN193616**		x
01508/7181	M3	766	**MN193617**		
01508/7182	M3	766	**MN193618**		
01508/7183	M3	766	**MN193619**		
01508/7184	M3	766	**MN193620**		
01508/7185	M3	766	**MN193621**		
01508/7186	M3	766	**MN193622**	**MN299118**	
01508/7187	M3	766	**MN193623**	**MN299119**	
01508/7188	M3	766	**MN193624**	**MN299120**	
01509/7189	M3	767	**MN193625**		x
01509/7190	M3	767	**MN193626**		
01509/7191	M3	767	**MN193627**		
01509/7192	M3	767	**MN193628**		
01509/7193	M3	767	**MN193629**		
01509/7194	M3	767	**MN193630**		x
01509/7195	M3	767	**MN193631**		
01509/7196	M3	767	**MN193632**		
01509/7197	M3	767	**MN193633**		
01509/7198	M3	767	**MN193634**		
01510/7199	M3	768	**MN193635**		
01510/7200	M3	768	**MN193636**		
01510/7201	M3	768	**MN193637**		x
01510/7202	M3	768	**MN193638**		
01510/7203	M3	768	**MN193639**		
01510/7204	M3	768	**MN193640**		x
01510/7205	M3	768	**MN193641**		
01510/7206	M3	768	**MN193642**		
01510/7207	M3	768	**MN193643**		
01510/7208	M3	768	**MN193644**		
01511/7209	M3	769	**MN193645**		x
01511/7210	M3	769	**MN193646**		
01511/7212	M3	769	**MN193647**		x
01075/7616	M5	444			x
01075/7617	M5	444			x
*Trochulus hispidus*					
01088/937	—	145			x
01088/939	—	145			x
01089/947	—	144			x
01135/1331	—	167	HQ204496		
01035/2000	—	237			x
01102/3144	—	319			x
01102/3147	—	319			x
