# Supplementary material for: Genome‐wide nuclear data confirm two species in the Alpine endemic land snail Noricella oreinos s.l. (Gastropoda, Hygromiidae)
Source: J Zool Syst Evol Res. 2020 Jan 24;58(4):982–1004. doi: 10.1111/jzs.12362 (PMC8609433; doi:10.1111/jzs.12362)
Supplement: Supplementary file 1 — Method S1. Sequence generation. Method S2. Increasing DNA concentration for AFLP generation. Method S3. Amplified fragment length polymorphism. Table S1. Primer combinations used for COI and ITS2 amplification. Table S2. List of the AFLP datasets. The number of mountain regions (Nreg), sampling localities (Nloc), specimens (N), and AFLP markers (NAFLP) is included with the applied programs or scripts (Splitstree, Past3, GenAlEx, AFLPdat.R, STRUCTURE). The number of K chosen for STRUCTURE analysis is given. The datasets D* and E1* were generated for AMOVA calculations by excluding localities with single samples from the datasets D and E. For hierarchical STRUCTURE cluster analyses, three subsets E2‐E4 were defined as specified in the text. Table S3. Genetic diversity calculated for complete AFLP dataset A and the two Noricella taxa (datasets D* and E1*). For each locality, the number of specimens N, the mean observed number of AFLP fragments N A, the percentage of polymorphic fragments PPF and the Nei’s gene diversity (D) are given. Figure S1. Neighbor‐joining tree inferred from mitochondrial COI sequences. Colors indicate Noricella o. scheerpeltzi (blue) and Noricella o. oreinos (pink). Bootstrap values >75% are given at the nodes. The scale bar represents 0.02 substitutions expected per aligned positions. One sequence of T. hispidus (black) was used as outgroup. Figure S2. Scatter plots of the first and second axis of the PCoA based on Jaccard similarity estimates calculated for the AFLP dataset A. In total, 50 individuals of Noricella o. scheerpeltzi (blue dots), 152 individuals of Noricella o. oreinos (pink dots), and six individuals of T. hispidus used as outgroup (black triangles) were analyzed. The axes are scaled by the square root of the Eigenvalues. Eigenvalues and variance represented by the first two axes are indicated at the axes (a) and eigenvalues of the first 10 axes are illustrated (b). Specimens of the potential contact zone (M6 region, Haller Mauer [file JZS-58-982-s002.pdf]

SUPPORTING INFORMATION

Genome-wide nuclear data confirm two species in the Alpine endemic land snail *Noricella oreinos* s.l. (Gastropoda, Hygromiidae)

Sonja Bamberger*, Michael Duda, Andreas Tribsch, Elisabeth Haring, Helmut Sattmann, Oliver Macek, Matthias Affenzeller, Luise Kruckenhauser

*corresponding author

- Supporting Information Method S1. Sequence generation
- Supporting Information Method S2. Increasing DNA concentration for AFLP generation
- Supporting Information Method S3. Amplified Fragment Length Polymorphism
- Supporting Information Table S1. Primer combinations for *COI* and *ITS2* amplification
- Supporting Information Table S2. List of the AFLP datasets
- Supporting Information Table S3. Genetic diversity calculated for the complete AFLP dataset A and the two *Noricella* taxa
- Supporting Information Fig. S1. Neighbor-joining tree inferred from mitochondrial *COI* sequences.
- Supporting Information Fig. S2. Scatter plots of the first and second axis of the PCoA based on Jaccard similarity estimates calculated for the AFLP dataset A.
- Supporting Information Fig. S3. Mean and SD values of the ln Pr(X|K) estimates and the ΔK values for each K as computed in STRUCTURE for the datasets B-D.
- Supporting Information Fig. S4. Mean and SD values of the ln Pr(X|K) estimates and the ΔK values for each K as computed in STRUCTURE for the datasets E1-E4.
- Supporting Information Data S1. *COI* alignment.
- Supporting Information Data S2. *ITS2* alignment.
- Supporting Information Data S3. AFLP dataset A.

Method S1

Sequence generation – Control reactions for DNA extraction and PCR amplification were included in mt *COI* amplification reactions to detect contaminations. The *COI* amplification reactions were performed following the QIAGEN TopTaq manufacturer's protocol with 1x TopTaq PCR Buffer, 1x Q-Solution™, 1.5 mM $MgCl_2$, 0.2 mM of each dNTP, 0.5 µM of each primer, 0.5 U TopTaq DNA polymerase, 1 µl template DNA and nuclease free water to a total volume of 25 µl. PCR conditions were: 94°C for 3 min, 35 cycles of 94°C for 30 sec, 54°C for 30 sec, 72°C for 30 sec, followed by 72°C for 7 min.

The *ITS2* sequences were amplified using the TopTaq DNA polymerase (Qiagen). In case of length polymorphisms or more than one allele at the *ITS2* locus, direct sequencing of the PCR was unsuccessful. PCR reactions were repeated from such specimens using the Q5® High-Fidelity DNA polymerase (NEB). The *ITS2* master mix included 1x Q5 Reaction Buffer, 0.2 mM of each dNTP, 0.5 µM of each primer, 0.5 U Q5 High-Fidelity DNA Polymerase, 0.5 µl template DNA and nuclease free water to a total volume of 25 µl. PCR conditions were: 98°C for 30 sec, 30 cycles of 98°C for 10 sec, 63°C for 20 sec, 72°C for 30 sec, followed by 72°C for 7 min. Subsequent cloning and sequencing was performed according to Kruckenhauser et al. (2014). In general, up to four clones per individual were sequenced. From the nine individuals which possessed the *ITS2* allele of both *N. oreinos* subspecies, DNA extraction and cloning of the *ITS2* fragment was repeated. Cloned sequences that occurred more than once for an individual were removed in the final dataset.

Method S2

Increasing DNA concentration for AFLP generation – If the concentration of available or newly-extracted DNA samples was below 10 ng/µl, the samples were post-processed: The AE-based DNA samples were precipitated using a conventional protocol, whereas samples, which had been eluted in nuclease free water, were concentrated using a vacuum centrifuge (UniEquip UniVapo 150 ECH). For precipitation, 1/10 Vol. 3M sodium acetate (pH 5.2), 9/10 Vol. DNA and 3x Vol. 96% ethanol were used according to a standard precipitation protocol. The pellet was dissolved in 15-30 µl nuclease free water (Qiagen). For the vacuum centrifuge concentration, DNA samples were incubated at 50°C for 3.25 hours and 15 µl sterile ddH_2O were added.

Method S3

Amplified Fragment Length Polymorphism – The various steps of restriction-ligation, PCR amplification and purification were carried out simultaneously for 48 samples in a GeneAmp® PCR System 9700 thermocycler (Thermo Fisher Scientific Inc.). Unless otherwise indicated, all chemicals

were purchased from VWR. The initial selective PCR primer pairs were chosen after a primer test using samples of 16 specimens and nine selective primer pair combinations with three selective nucleotides each (data not shown).

For each reaction, 5 µl of template DNA (minimum of 50 ng DNA) was digested for three hours at 37 °C with 6 µl of the corresponding master mix. The master mix contained 1x T4 Ligase Buffer (Promega), 50 mM NaCl, 0.55 µg BSA (Promega), 4.55 µM *Mse*I adaptor (MWG Eurofins, Germany), 0.46 µM *EcoR*I adaptor (MWG Eurofins), 1 U *Mse*I enzyme (Promega), 8 U *EcoR*I enzyme (Promega), 0.9 U T4 DNA Ligase (Promega) and sterile ddH$_2$O. Ten percent of the DNA samples were selected randomly and repeated from the restriction-ligation step onwards to test the repeatability of the subsequently scored AFLP fragments and to estimate the error rate. Additionally, each of the six restriction ligation plates contained a control reaction with no template and one sample chosen for repetition.

The pre-selective amplification reactions were performed using PCR primer pairs with one selective base at the 3´-end of each primer. The 10 µl reaction consisted of 2 µl of the 20-fold diluted restriction-ligation template, 1x Green GoTaq buffer (Promega), 0.22 mM dNTPs, 0.29 µM *EcoR*I-A primer (MWG Eurofins), 0.29 µM *Mse*I-C primer (MWG Eurofins) and 0.125 U GoTaq G2 (Promega). Pre-selective PCR conditions were as follows: 72°C for 2 min, 30 cycles of 94°C for 30 sec, 56°C for 30 sec, and 72°C for 1 min, finishing with 72°C for 10 min.

For amplification in selective PCR reactions, primer pairs with three selective bases at the 3´-end of each PCR primer were used. The following combinations of fluorophore-labelled *EcoR*I primers and unlabelled *Mse*I primers were used: *EcoR*I-ATG$^{6\text{-}FAM}$/*Mse*I-CTT, *EcoR*I-AGG^{VIC}/*Mse*I-CTC and *EcoR*I-ACA^{NED}/*Mse*I-CAA. The selective amplification was carried out within 10 µl of 1x Green GoTaq buffer, 0.22 mM dNTPs, each 0.27 µM of the respective *EcoR*I-NNN primer (Applied Biosystems) and *Mse*I-NNN primer (MWG Eurofins), 0.2 U GoTaq G2, sterile ddH$_2$O and 2 µl of the 20-fold diluted pre-selective PCR product. For all three primer pairs, selective amplification was performed with the following cycling conditions: 94°C for 2 min, 10 cycles of 94°C for 20 sec, 66°C (decreasing 1°C in each cycle) for 30 sec, and 72°C for 2 min, followed by 20 cycles of 94°C for 20 sec, 56°C for 30 sec, and 72°C for 2 min, and a final step of 60°C for 30 min.

Following the protocol of (Bendiksby, Tribsch, Borgen, Trávníček, & Brysting, 2011), 6 µl of the NED-labelled PCR products were pooled with both 3 µl of FAM and VIC-labelled product and purified using Sephadex^{TM} G-50 Superfine resins (GE Healthcare BioSciences). For MegaBACE preparation, 12.9 µl AD, 0.1 µl ET-ROX 400-R MegaBACE size standard and 2 µl (in case of the first run) or respective 1.6 µl of the purified PCR products were denatured at 95°C for 3 min and immediately cooled on ice until capillary electrophoresis.

Table S1. Primer combinations for *COI* and *ITS2* amplification.

Primer 5'-3'	Origin	Fragment size (bp)
***COI*folmerfwd:** GGTCAACAATCATAAAGATATTGG	Duda et al. (2011) modified from Folmer et al. (1994)	705
***COI*schneckrev:** TATACTTCTGGATGACCAAAAAATCA	Duda et al. (2011), modified from Gittenberger et al. (2004)	
**LSU-1:** CTAGCTGCGAGAATTAATGTGA	Wade & Mordan (2000)	up to 954
**LSU-3:** ACTTTCCCTCACGGTACTTG	Wade & Mordan (2000)	

Table S2. List of the AFLP datasets. The number of mountain regions (Nreg), sampling localities (Nloc), specimens (N) and AFLP markers (N_{AFLP}) are included with the applied programs or scripts (Splitstree, Past3, GenAlEx, AFLPdat.R, STRUCTURE). The number of K chosen for STRUCTURE computations is given. The datasets D* and E1* were generated for AMOVA calculations by excluding localities with single samples from the datasets D and E. For hierarchical STRUCTURE cluster analyses, three subsets E2-E4 were generated as specified in the text.

Dataset	Nreg	Nloc	N	N_{AFLP}	Splitstree	PAST3	GenAlEx	AFLPdat.R	STRUCTURE
N. oreinos, *T. hispidus* (outgroup)									
dataset A	-	36	208	329		X			
N. oreinos – complete dataset (mountain regions M2-M15)									
dataset B	14	32	202	316	X	X			1-20
dataset B*	14	29	199	316			X	X	
Supposed contact zone (M5, M6) of *N. oreinos* subspecies and adjacent mountain (M8)									
dataset C	3	7	114	292					1-7
N. o. scheerpeltzi (M2-M5)									
dataset D	4	15	50	263		X			1-10
dataset D*	4	13	48	261			X	X	
N. o. oreinos (M6-M15)									
dataset E1	10	17	152	293		X			1-15
dataset E1*	10	16	151	293			X	X	
N. o. oreinos – dataset E1 without Haller Mauern (M7-M15)									
dataset E2	9	13	72	274					1-15
N. o. oreinos – Gesäuse mountain regions (M7-M10)									
dataset E3	4	5	43	247					1-7
N. o. oreinos – Hochschwab to Schneeberg (M11-M15)									
dataset E4	5	8	29	238					1-7

Table S3. Genetic diversity calculated for the complete AFLP dataset A and the two *Noricella* taxa (datasets D* and E1*). For each locality, the number of specimens N, the mean observed number of AFLP fragments N_A, the percentage of polymorphic fragments PPF and the Nei's gene diversity (D) are given.

Locality	N	N_A	PPF	D
N. oreinos	199	1.94	93.7	
N. o. scheerpeltzi	48	1.81	81.2	
382	2	0.66	6.1	0.061
383	2	0.77	13.4	0.134
389	2	0.65	5.0	0.050
387	2	0.61	2.3	0.023
765	5	0.86	17.2	0.081
766	2	0.77	10.0	0.100
767	2	0.70	8.8	0.088
768	2	0.81	12.3	0.123
769	2	0.79	14.9	0.149
369	4	0.87	18.8	0.104
367	4	0.80	14.9	0.082
443	12	1.18	40.6	0.128
444	7	1.12	36.8	0.137
Localities mean	4	0.81	15.5	
SE	0.0	0.01	3.2	

Locality	N	N_A	PPF	D
N. o. oreinos	151	1.83	82.6	
783	18	1.01	28.3	0.076
782	23	1.13	35.8	0.083
784	11	1.11	33.1	0.101
785	28	1.06	32.8	0.076
55	5	0.85	17.1	0.072
665	6	0.92	22.2	0.071
781	15	1.06	32.1	0.090
779	10	0.87	21.8	0.076
399	7	0.89	21.8	0.085
134	7	0.98	29.7	0.126
588	3	0.71	13.0	0.086
338	8	0.81	19.8	0.074
737	2	0.68	7.2	0.072
79	4	0.67	8.5	0.046
448	2	0.60	4.1	0.041
178	2	0.63	5.8	0.058
Localities mean	9	0.87	20.8	
SE	0.1	0.01	2.7	

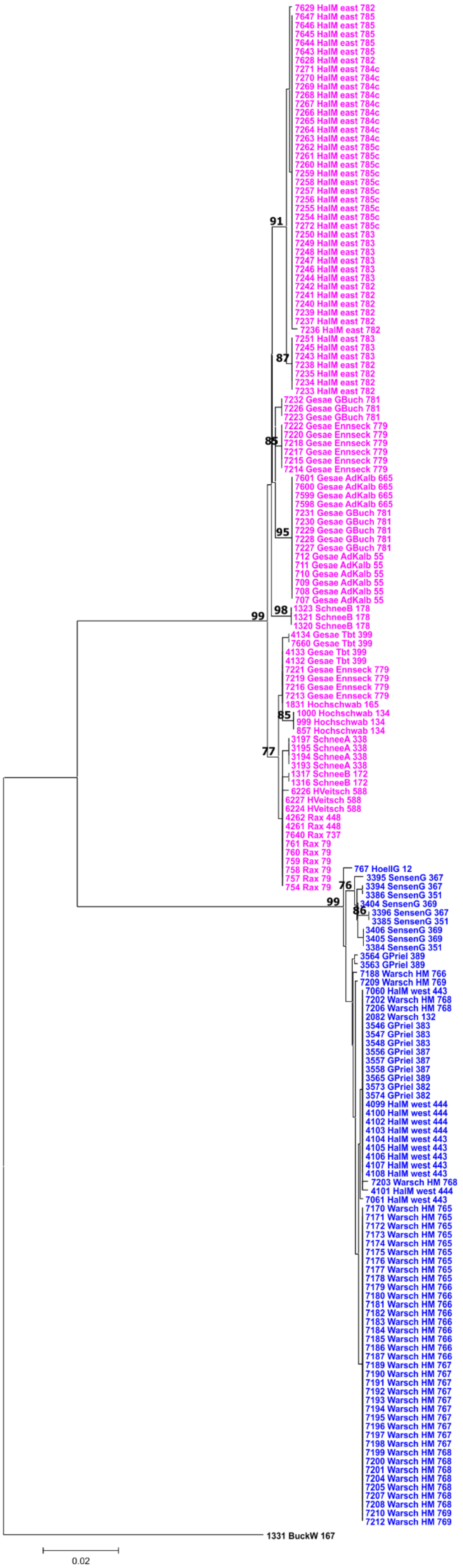

Fig. S1. Neighbor-joining tree inferred from mitochondrial *COI* sequences. Colours indicate *N. o. oreinos* (pink) and *N. o. scheerpeltzi* (blue). Bootstrap values >75% are given at the nodes. The scale bar represents 0.02 substitutions expected per aligned positions. One sequence of *T. hispidus* (black) was used as outgroup.

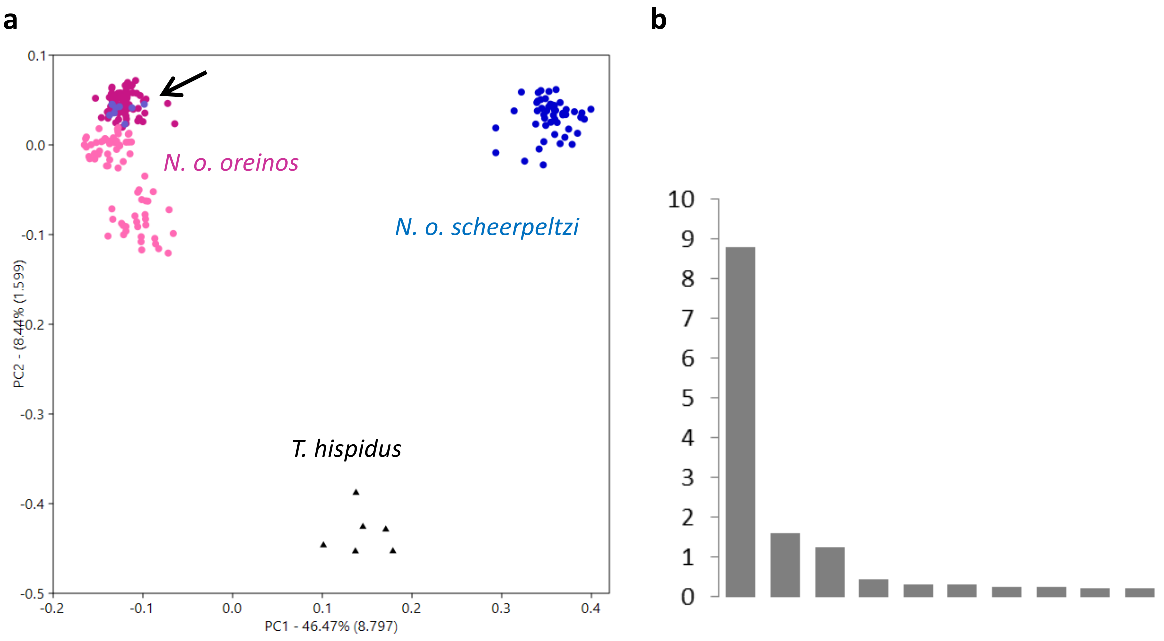

Fig. S2. Scatter plots of the first and second axis of the PCoA based on Jaccard similarity estimates calculated for the dataset A. In total, 152 specimens of *N. o. oreinos* (pink dots), 50 specimens of *N. o. scheerpeltzi* (blue dots) and six specimens of *T. hispidus* used as outgroup (black triangles) were analysed. The axes are scaled by the square root of the Eigenvalues. Eigenvalues and variance represented by the first two axes are indicated at the axes (a) and Eigenvalues of the first ten axes are illustrated (b). Specimens of the potential contact zone (M6 region, Haller Mauern east) are drawn with dark pink dots. The specimens found heterozygous, possessing the *ITS2* alleles of both taxa (violet dots), are clustering within the M6 region (black arrow).

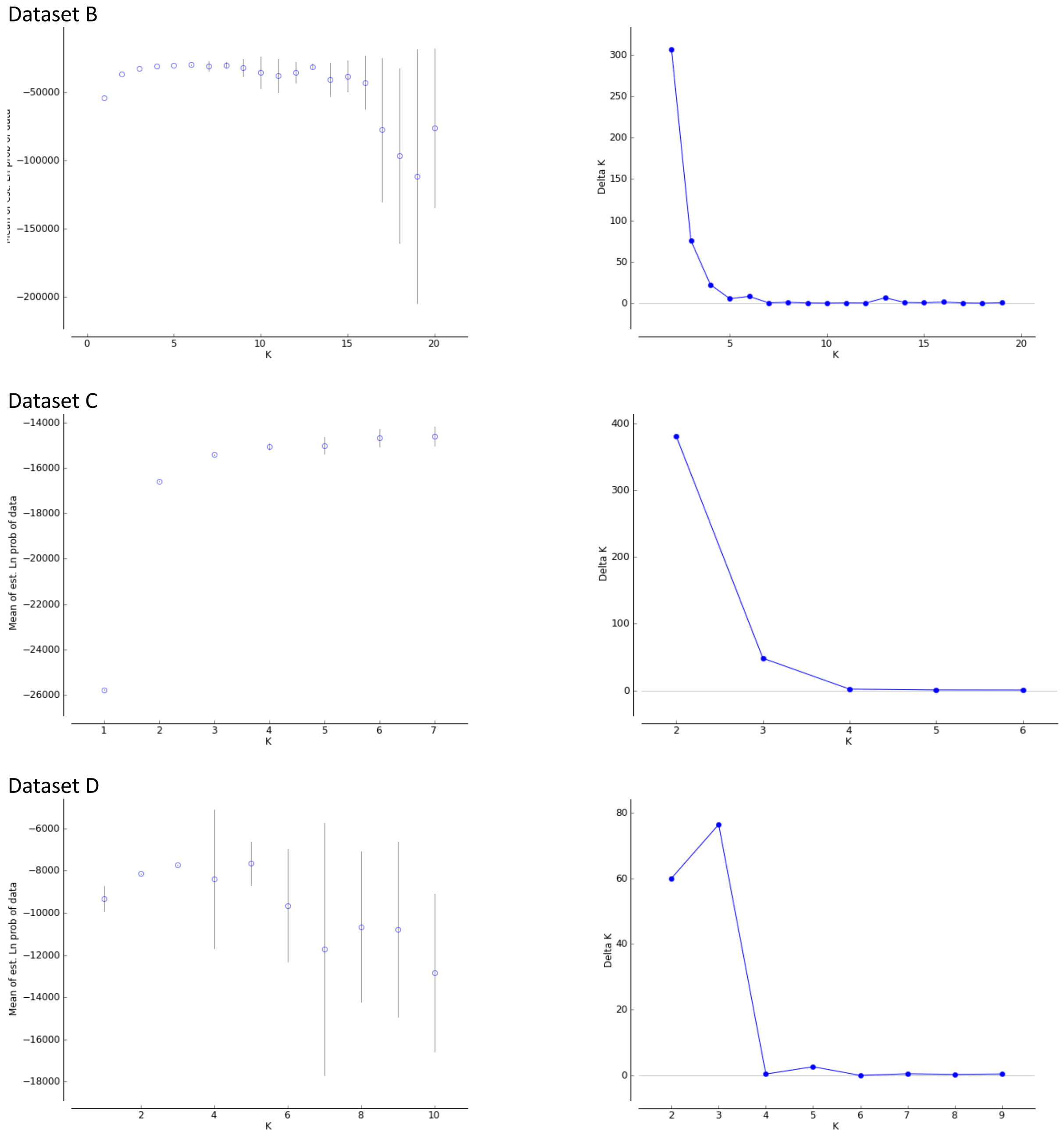

Fig. S3. Mean and SD values of the ln Pr(X|K) estimates and the ΔK values for each K as computed in STRUCTURE for the datasets B-D.

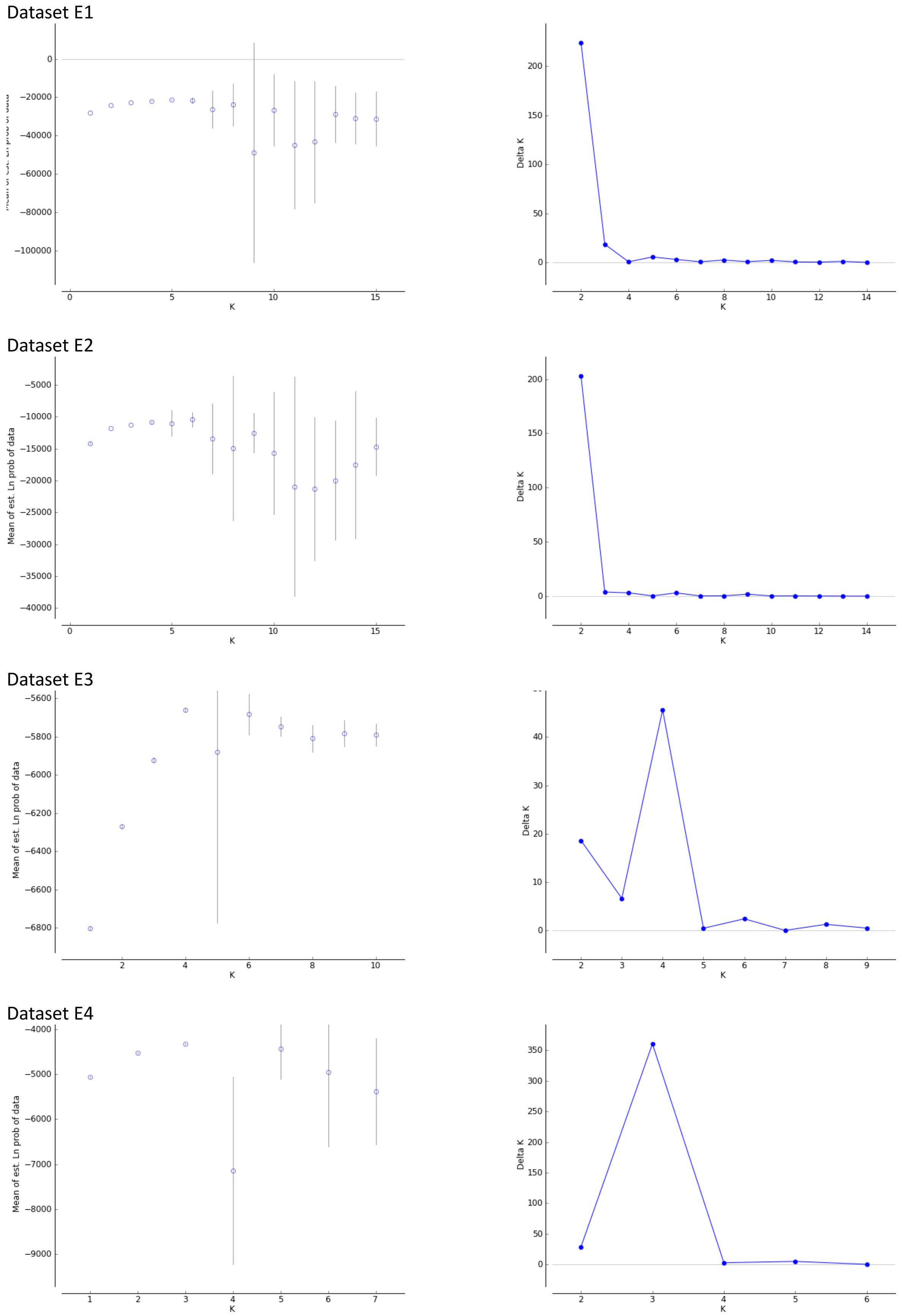

Fig. S4. Mean and SD values of the ln Pr(X|K) estimates and the ΔK values for each K as computed in STRUCTURE for the datasets E1-E4.

References

Bendiksby, M., Tribsch, A., Borgen, L., Trávníček, P., & Brysting, A. K. (2011). Allopolyploid origins of the *Galeopsis* tetraploids - revisiting Müntzing's classical textbook example using molecular tools. *New Phytologist*, 191(4), 1150–1167. https://doi.org/10.1111/j.1469-8137.2011.03753.x

Duda, M., Sattmann, H., Haring, E., Bartel, D., Winkler, H., Harl, J., & Kruckenhauser, L. (2011). Genetic differentiation and shell morphology of differentiation in the *Trochulus oreinos* (Wagner, 1915) and *T. hispidus* (Linnaeus, 1758) (Pulmonata: Hygromiidae) in the northeastern Alps. *Journal of Molluscan Studies*, 77(1), 30–40. https://doi.org/10.1093/mollus/eyq037

Folmer, O., Black, M., Hoeh, W., Lutz, R., & Vrijenhoek, R. (1994). DNA primers for amplification of mitochondrial *cytochrome c oxidase subunit I* from diverse metazoan invertebrates. *Molecular Marine Biology and Biotechnology*, 3(5), 294–299.

Gittenberger, E., Piel, W. H., & Groenenberg, D. S. J. (2004). The Pleistocene glaciations and the evolutionary history of the polytypic snail species *Arianta arbustorum* (Gastropoda, Pulmonata, Helicidae). *Molecular Phylogenetics and Evolution*, 30(1), 64–73. https://doi.org/10.1016/S1055-7903(03)00182-9

Kruckenhauser, L., Duda, M., Bartel, D., Sattmann, H., Harl, J., Kirchner, S., & Haring, E. (2014). Paraphyly and budding speciation in the hairy snail (Pulmonata, Hygromiidae). *Zoologica Scripta*, 43(3), 273–288. https://doi.org/10.1111/zsc.12046

Wade, C. M., & Mordan, P. B. (2000). Evolution within the gastropod molluscs; using the ribosomal RNA gene-cluster as an indicator of phylogenetic relationships. *Journal of Molluscan Studies*, 66(4), 565–570. https://doi.org/10.1093/mollus/66.4.565
